# Design- and Voxel-Based Analysis of a Topology–Density Dual-Gradient TPMS Absorber for Impact-Load Mitigation

**DOI:** 10.3390/ma19183904

**Published:** 2026-09-14

**Authors:** Wenying Xu, Jiawei Xu, Yonglin Chen, Dongyu Fan, Yongbin Wang, Tao Yu, Siyu Chen, Weidong Yang

**Affiliations:** 1 School of Aerospace Engineering and Applied Mechanics, Tongji University, Shanghai 200092, China; 2311376@tongji.edu.cn (W.X.); chenyonglin@tongji.edu.cn (Y.C.); 20501@tongji.edu.cn (W.Y.); 2 Beijing Institute of Space Mechanics & Electricity, Beijing 100094, China

**Keywords:** triply periodic minimal surface, topology–density dual gradient, voxel-based finite element modeling, impact energy absorption, peak-stress mitigation, aerospace buffer

## Abstract

**Highlights:**

- A 0.33 mm C3D8R voxel model achieved 1.11% density error and reduced runtime by 88%.
- IWP20 and P40 showed complementary compression and energy-absorption characteristics.
- Topology-density grading promoted sequential crushing and delayed densification.
- At 125 J, TDDG reduced peak stress by 32.2% versus U-P30 and 24.6% versus DG-P20-P40.
- TDDG retained peak-stress mitigation across impact energies from 62.5 to 160 J.

**Abstract:**

Triply periodic minimal surface (TPMS) lattices are promising lightweight impact absorbers, but high-fidelity finite element modeling is costly, and uniform designs can develop high stress peaks during densification. This study presents a voxel-based finite element framework and a topology–density dual-gradient (TDDG) TPMS absorber. A convergence study selected a voxel size of 0.33 mm, yielding a 1.11% relative-density error and converged mechanical responses. Compared with a converged C3D4 tetrahedral model, the C3D8R voxel model reduced wall-clock time from 4222 to 497 s. Following validation against quasi-static compression tests, P, G, and IWP topologies at 20%, 30%, and 40% relative densities were screened. IWP20 and P40 were assigned to the impact- and support-facing regions and connected by normalized sigmoid interpolation. Under a 125 J impact, simulations predicted that TDDG-IWP20-P40 reduced peak nominal impact stress by 32.2% and 24.6% relative to U-P30 and DG-P20-P40, respectively, while maintaining comparable SEA. Additional simulations at 62.5 and 160 J confirmed lower peak stress than U-P30, with the added benefit over density-only grading becoming more pronounced at higher impact energy. Progressive crushing and delayed densification demonstrate the potential of TDDG TPMS absorbers for impact-load mitigation and future aerospace buffer designs.

## 1. Introduction

Spacecraft landing systems require lightweight buffer structures capable of reducing transmitted loads during touchdown while maintaining sufficient energy absorption capacity [1,2,3,4]. During landing, the absorber must dissipate kinetic energy within a limited stroke and suppress peak stress or acceleration transmitted to the protected structure [5,6]. Conventional landing buffer designs, including crushable honeycombs, metallic foams, thin-walled tubes, and shock absorbers, have been widely investigated for aerospace and planetary landing applications [7,8]. However, these structures often face trade-offs among mass efficiency, stable collapse, controllable deformation, and manufacturability, especially when the available volume and allowable peak load are constrained.

Additively manufactured lattice structures offer a new design space for aerospace energy absorbers because their stiffness, strength, and collapse modes can be adjusted through topology and relative density [9,10,11,12,13,14,15]. Compared with stochastic foams and conventional honeycombs, architected lattices provide better geometric repeatability and more predictable mechanical behavior [16,17,18]. Recent studies have shown that lattice and cellular metamaterials can achieve high specific stiffness, high specific strength, programmable deformation, and multifunctional performance, making them attractive for aerospace structures where lightweight design and impact resistance are both required [19,20,21,22].

Among various lattice topologies, triply periodic minimal surface (TPMS) structures have attracted increasing attention because of their smooth continuous surfaces, high surface-to-volume ratio, controllable pore morphology, and absence of sharp strut intersections [23,24,25,26,27,28,29]. Typical TPMS topologies, such as Schwarz primitive (P), Gyroid (G), and I-graph and wrapped package (IWP), can be generated from implicit functions and fabricated by additive manufacturing. Their mechanical response can be tailored by changing the unit cell size, relative density, wall thickness, and loading direction [30,31,32,33]. Compared with beam-based lattices, sheet-based TPMS structures generally provide smoother stress transfer and improved deformation stability, which are beneficial for compression and impact energy absorption [34,35].

Functionally graded lattice structures further improve the design flexibility of energy absorbers by spatially varying density or topology. In graded structures, low-density regions can deform first and reduce the initial impact force, whereas high-density regions can provide late-stage support and delay complete densification [36,37,38,39]. However, most existing TPMS energy absorption studies focus on single-topology or density-only graded designs. The potential of hybrid graded structures that combine different TPMS topologies at different density regions remains insufficiently explored, especially for impact attenuation where the redistribution of energy absorption over time is critical.

Reliable finite element modeling is also essential for TPMS impact analysis. STL-based TPMS geometries usually contain complex curved surfaces and large numbers of triangular facets, which may lead to time-consuming tetrahedral meshing and unstable contact behavior during large deformation. Voxel-based modeling provides an alternative by converting complex geometries into structured hexahedral meshes, which can simplify mesh generation and improve computational efficiency [40,41]. Nevertheless, the accuracy and efficiency of voxelized TPMS models must be evaluated through convergence analysis and comparison with conventional mesh strategies.

To address the computational cost of high-fidelity TPMS modeling and the limited load-regulation capability of uniform or single-topology graded absorbers, this study proposes a voxel-based design and simulation framework for topology–density dual-gradient (TDDG) TPMS structures. The framework combines structured-hexahedral voxel modeling, quasi-static validation and constituent screening, and dynamic impact analysis. The density-dependent responses of the P, G, and IWP topologies are first evaluated to identify configurations with complementary deformation and energy-absorption characteristics. Based on this screening, a TDDG-IWP20-P40 absorber is constructed using a normalized sigmoid transition in both topology and relative density. Its impact response is compared with those of the uniform U-P30 and single-topology density-graded DG-P20-P40 structures to determine whether coordinated topology and density grading can mitigate peak impact stress while maintaining energy-absorption capacity. Particular attention is given to the relationship between progressive crushing, densification, and impact-load regulation. A conceptual cylindrical buffer core is also presented to examine the geometric transferability of the proposed architecture to aerospace buffer applications.

## 2. TPMS Design and Computational–Experimental Methodology

### 2.1. TPMS Geometric Design and Screening Matrix

In this study, triply periodic minimal surface structures were chosen as the geometric foundation of the energy-absorbing lattices because of their continuous surfaces, periodic topology, and high geometric controllability. Three representative sheet-based TPMS topologies—P, G, and IWP—were adopted for this purpose. These topologies were screened at different relative densities to identify suitable topology–density combinations for the subsequent dual-gradient design.

The implicit functions for the P, G, and IWP surfaces are defined as follows:

(1) P:cosX+cosY+cosZ=C

(2) G:sinXcosY+sinYcosZ+sinZcosX=C

(3) $$\begin{array}{cc} \mathrm{IWP}: & 2\left(\cos X\cos Y+\cos Y\cos Z+\cos Z\cos X\right)\\ & -\left(\cos2X+\cos2Y+\cos2Z\right)=C \end{array}$$

where *C* is the level-set parameter that controls the position of the surface. The normalized spatial coordinates *X*, *Y* and *Z* are defined as follows:

(4) X=2πx/l, Y=2πy/l, Z=2πz/l

where *l* is the unit cell size. *x*, *y*, *z* are the Cartesian coordinates.

A sheet-based TPMS formulation was adopted in this study. Instead of using an infinitely thin mathematical surface, the sheet-based solid domain was generated by retaining the region between two level sets, *C*_1_ and *C*_2_:

(5) $$C_{1}\leq F\left(x,y,z\right)\leq C_{2}$$

where *F*(*x*, *y*, *z*) is the corresponding TPMS implicit function. The relative density of the TPMS lattice was controlled by adjusting the interval between *C*_1_ and *C*_2_, where *C*_1_ and *C*_2_ were set as opposite values. Each pair of *C*_1_ and *C*_2_ therefore corresponded to a specific relative density.

**Figure 1:**
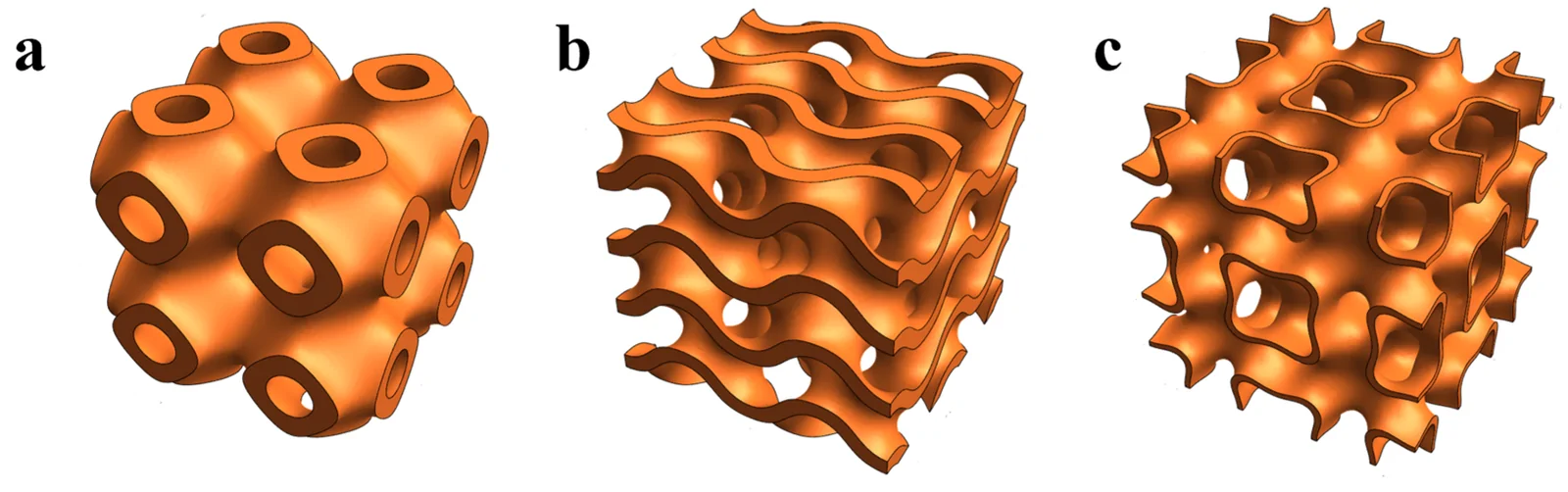
Three types of TPMS structures generated by the implicit function method: (**a**) P; (**b**) G; (**c**) IWP.

To identify a suitable topology for subsequent hybrid graded design, three representative sheet-based TPMS structures were investigated, namely the P, G, and IWP topologies, as shown in Figure 1. For each topology, homogeneous specimens with relative densities of 20%, 30%, and 40% were generated, resulting in nine simulation groups in total. All specimens had the same geometric dimensions, with a unit cell size of 10 mm, a 3 × 3 × 3 periodic array, and an overall size of 30 mm × 30 mm × 30 mm.

The topology screening matrix is summarized in Table 1. The screening was conducted to compare the stiffness, initial peak stress, energy absorption efficiency, and densification behavior of the nine topology–density combinations and to identify suitable candidates for the impact- and support-side regions of the dual-gradient absorber.

### 2.2. Voxel-Based Numerical Modeling Framework

#### 2.2.1. STL-to-INP Voxelization Workflow

**Figure 2:**
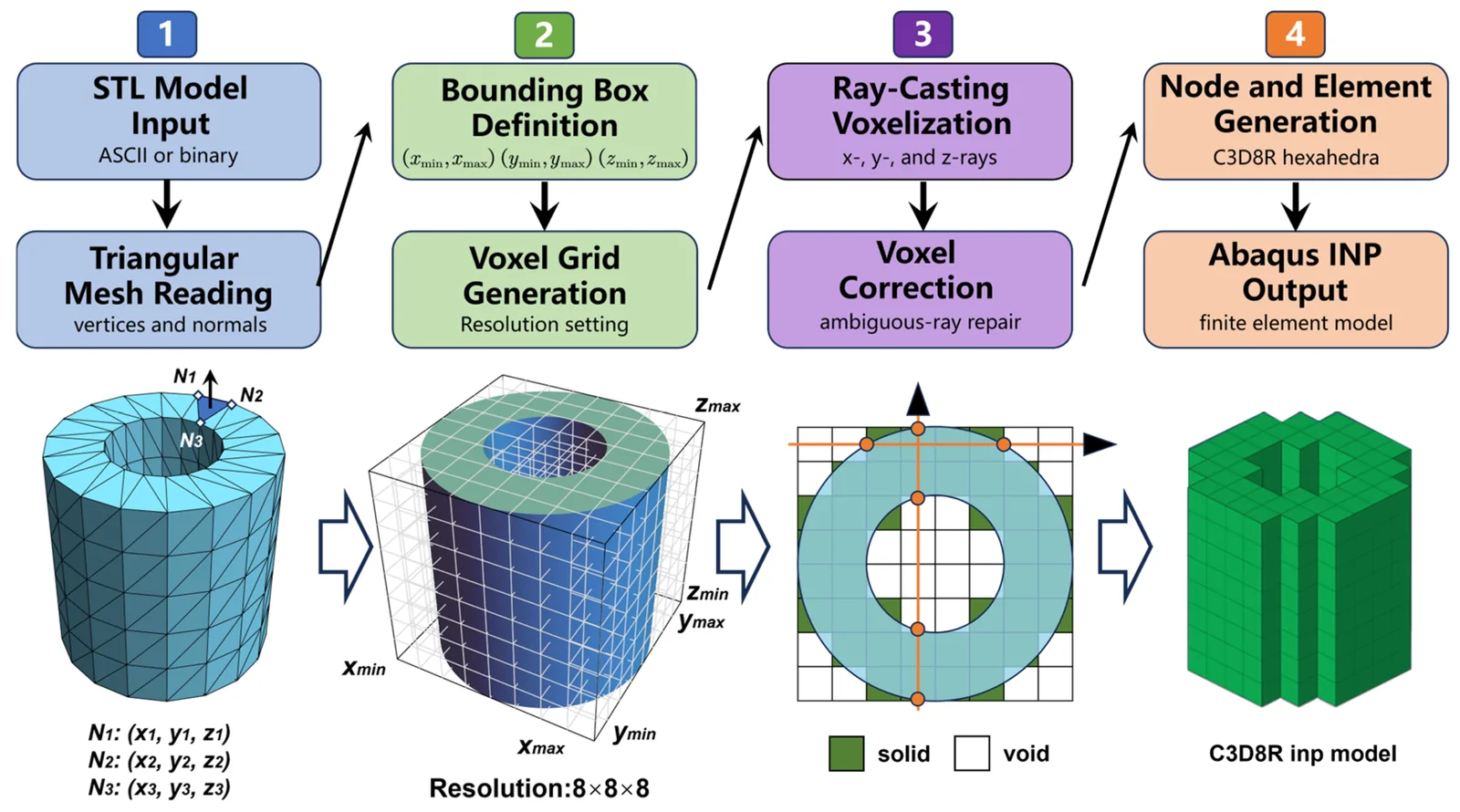
Workflow for converting STL-based geometries into voxelized Abaqus finite element models.

The generated TPMS geometries were first exported as STL files, and the triangular facet models in the STL files were subsequently converted into voxel-based finite element INP models using an in-house Python (v2024.1) program, as shown in Figure 2. The purpose of the voxelization procedure was to transform complex TPMS structures into structured hexahedral meshes that could be directly imported into Abaqus (v2021) for compression and impact simulations.

The program first determined whether the STL file was in ASCII or binary format, and then extracted the vertex coordinates and facet normal vectors using the corresponding reader. The vertex coordinates of the triangular facets were then used to define the geometric boundary of the TPMS structure.

After reading the STL geometry, the bounding box of the model was calculated. For a model with bounding box dimensions *L_x_* = *x*_max_ − *x*_min_, *L_y_* = *y*_max_ − *y*_min_, and *L_z_* = *z*_max_ − *z*_min_, a regular Cartesian voxel grid was generated within the bounding box. The voxel resolution in the x, y, and z directions was prescribed as *N_x_*, *N_y_*, and *N_z_*, respectively. The corresponding voxel sizes were defined as

(6) $$\Delta x=\frac{L_{x}}{N_{x}},\;\Delta y=\frac{L_{y}}{N_{y}},\;\Delta z=\frac{L_{z}}{N_{z}}$$

Each voxel was represented by its center point,

(7) $$P_{ijk}=\left(x_{\min}+\left(i+\frac{1}{2}\right)\Delta x,\;y_{\min}+\left(j+\frac{1}{2}\right)\Delta y,\;z_{\min}+\left(k+\frac{1}{2}\right)\Delta z\right)$$

where *i* = 0, …, *N_x_* − 1, *j* = 0, …, *N_y_* − 1, and *k* = 0, …, *N_z_* − 1. Each voxel was then classified as solid or void according to its position relative to the closed STL surface.

A ray-casting method was used for the solid-void classification. Taking ray casting along the z-direction as an example, a ray was defined at each grid location as

(8) $$r\left(z\right)=\left(x_{i},\;y_{i},\;z\right)$$

Before calculating the exact intersection, triangular facets were first filtered according to their projected coordinate ranges. A triangular facet was considered a candidate intersecting facet only when

(9) $$x_{\min}^{t}\leq x_{i}\leq x_{\max}^{t},\;y_{\min}^{t}\leq y_{j}\leq y_{\max}^{t}$$

where $x_{\min}^{t}$, $x_{\max}^{t}$, $y_{\min}^{t}$ and $y_{\max}^{t}$ are the projected coordinate bounds of the triangular facet. For each candidate triangular facet, the projected point $\left(x_{i},y_{j}\right)$ was further checked using a point-in-triangle criterion. In barycentric form,

(10) $$\left(x_{i},y_{j}\right)=\lambda_{1}\left(x_{1},y_{1}\right)+\lambda_{2}\left(x_{2},y_{2}\right)+\lambda_{3}\left(x_{3},y_{3}\right)$$

with

(11) $$\lambda_{1}+\lambda_{2}+\lambda_{3}=1$$

The ray was regarded as intersecting the triangular facet when

(12) $$\lambda_{1}\geq 0,\;\lambda_{2}\geq 0,\;\lambda_{3}\geq 0$$

The *z*-coordinate of the intersection point was then calculated from the plane equation of the triangular facet,

(13) Ax+By+Cz+D=0

as

(14) $$z_{int}=\frac{-D-Ax_{i}-By_{j}}{C}$$

All intersection points along the ray were sorted in ascending order as

(15) $$z_{1}<z_{2}<\ldots <z_{n}$$

For a closed STL surface, the number of intersections *n* should be even, and the material regions along the ray were defined by paired intersections,

(16) $$\left(z_{1},z_{2}\right),\left(z_{3},z_{4}\right),\ldots ,\left(z_{n-1},z_{n}\right)$$

Accordingly, a voxel was classified as solid when its center coordinate *z_k_* satisfied

(17) $$z_{2q-1}<z_{k}<z_{2q},\;q=1,2,\ldots ,\frac{n}{2}$$

The same procedure was also applied along the other coordinate directions, and the results from multiple ray-casting directions were combined to reduce direction-dependent errors in complex TPMS regions.

When a ray produced an odd number of intersections, which may occur when the ray passes near a triangle edge or vertex or because of numerical tolerance, the ray was regarded as ambiguous. These ambiguous rays were corrected using the voxel states of the eight neighboring rays. At each voxel layer, the ambiguous voxel was marked as solid when at least half of its neighboring voxels were solid. This correction reduced isolated voxel errors and improved the continuity of the voxelized geometry.

After voxelization, the solid voxel matrix was converted into a finite element mesh. Each solid voxel was converted into one eight-node hexahedral element, and shared nodes between adjacent voxels were merged automatically. The nodal coordinates and element connectivity were then written to an Abaqus input file. The element type used for the voxel-based finite element model was C3D8R.

Although the voxelized representation introduces staircase-like discretization along curved TPMS boundaries, its influence on relative density and mechanical response was assessed through the voxel-size convergence analysis presented in Section 3.1.

#### 2.2.2. Finite Element Model and Analysis Settings

The voxelized TPMS models were imported into Abaqus for finite element analysis positioned between two rigid platens. The lower platen was fully constrained, whereas the upper platen was prescribed a displacement for quasi-static compression or assigned an initial velocity for impact loading.

Contact interactions were defined between the TPMS structure and the rigid platens, together with self-contact within the TPMS structure to account for contact between opposing surfaces during large deformation and progressive densification. Hard contact was adopted in the normal direction, while tangential behavior was described using a penalty-based friction formulation. The coefficients of friction were set to 0.2 for the platen–structure interfaces and 0.3 for TPMS self-contact.

The lower rigid platen was fully constrained. The upper platen was constrained against lateral translation and rotation and was allowed to move only along the loading direction. A downward displacement of 20 mm, corresponding to an engineering compressive strain of 66.7%, was prescribed using a smooth-step amplitude over a total analysis time of 0.2 s. The loading rate was selected such that the kinetic energy remained below 5% of the internal energy over the main compression stage.

**Figure 3:**
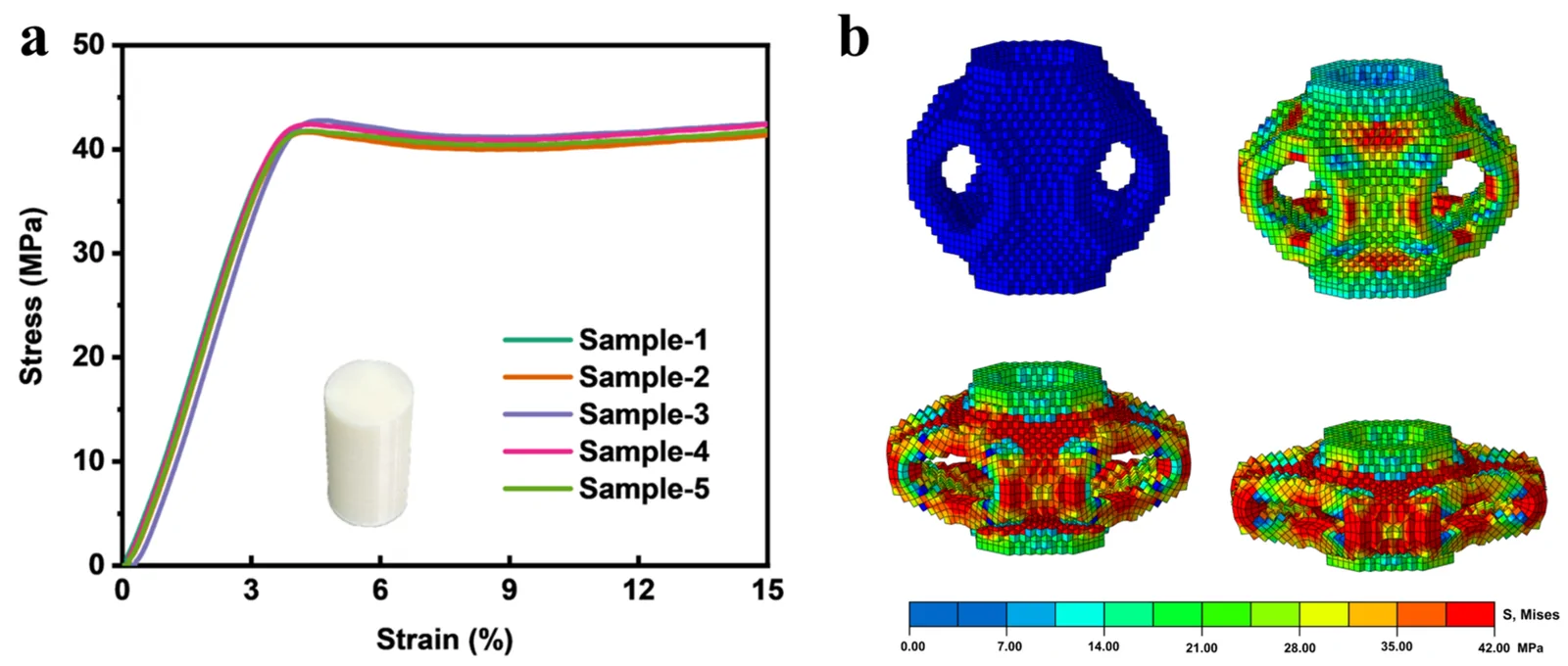
Finite Element Model Setup: (**a**) PLA material parameter testing; (**b**) stress contours of P structure with a relative density of 20% at 0%, 10%, 30%, and 50% compression.

The material employed in the finite element model was polylactic acid (PLA), modeled as an isotropic elastic-perfectly plastic material using the von Mises yield criterion. The elastic modulus, Poisson’s ratio, and initial yield stress are 1324 MPa, 0.30, and 42 MPa, respectively, which were obtained from compression tests on standard specimens, as shown in Figure 3. This simplified model was adopted to balance computational efficiency and global response prediction in the voxel-based simulations, but it does not explicitly account for strain-rate sensitivity, hardening, damage/failure, or printing-induced anisotropy of FFF-printed PLA.

To ensure methodological consistency across the different simulation stages, the same material constitutive model, contact formulations, and voxel-based discretization procedure were employed in both the quasi-static topology screening and subsequent impact analyses. The quasi-static simulations were used to characterize and compare the fundamental compressive responses of the TPMS topologies, whereas the impact simulations evaluated their energy-absorption behavior at a representative landing-impact velocity. This unified modeling framework minimized numerical variability associated with mesh generation and facilitated direct comparison across all structural configurations.

### 2.3. Specimen Fabrication and Quasi-Static Compression Tests

The TPMS lattice specimens were fabricated using fused filament fabrication with a Bambu H2D 3D printer (Bambu Lab, Shenzhen, China). Bambu PLA Basic filament was used as the printing material. Each specimen consisted of a 3 × 3 × 3 unit-cell array, with a unit cell size of 10 mm, resulting in an overall nominal dimension of 30 mm × 30 mm × 30 mm. The nozzle diameter was 0.4 mm, and the layer height was set to 0.12 mm. The printing speed and nozzle temperature were 200 mm/s and 220 °C, respectively. The solid regions of the specimens were printed using a 100% rectilinear infill strategy, and no support structures were used during fabrication.

After printing, the mass and external dimensions of the specimens were measured. The average masses of the specimens with relative densities of 20%, 30%, and 40% were 5.52 g, 8.68 g, and 11.91 g, respectively. Three specimens were fabricated for each design group to evaluate repeatability.

Quasi-static compression tests were conducted using an FR-103C computer-controlled universal testing machine equipped with a 50 kN load cell. The specimens were compressed between two parallel plates at a constant loading speed of 2 mm/min. No lubricant was applied between the specimen and the compression platens. The force and displacement data were recorded at a sampling rate of 2000 points per minute. For each group, three repeated compression tests were performed. The engineering stress was calculated by dividing the measured force by the initial cross-sectional area of the specimen, and the engineering strain was obtained from the platen displacement divided by the initial specimen height.

### 2.4. Performance Evaluation Metrics

#### 2.4.1. Quasi-Static Compression Metrics

**Figure 4:**
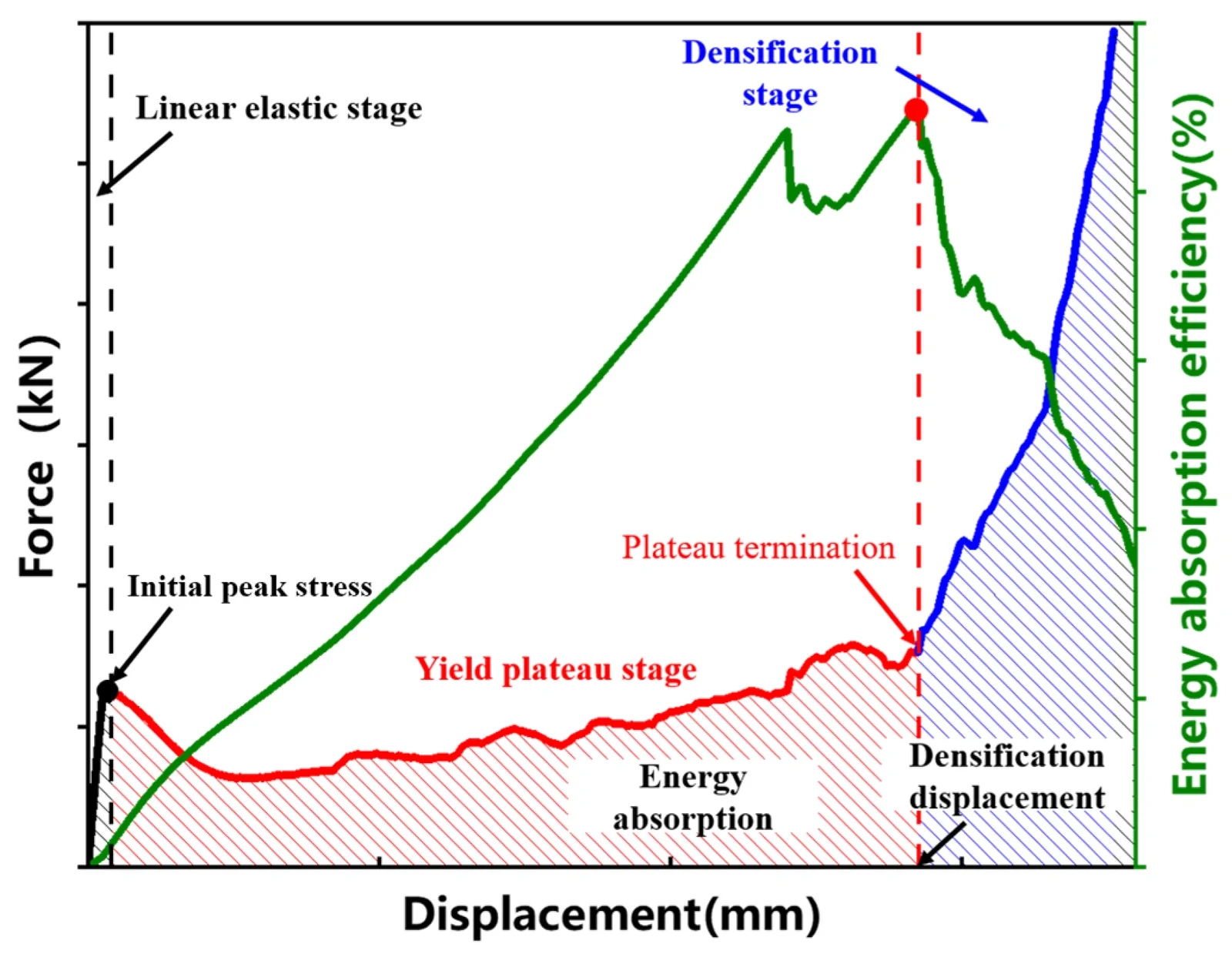
Schematic definitions of the quasi-static compression and energy-absorption metrics.

The quasi-static compression metrics adopted in this study are schematically defined in Figure 4. The initial stiffness was determined from the slope of the initial linear portion of the force–displacement curve. The peak compression force (*PCF*) was defined as the maximum force attained prior to the onset of densification.

The energy absorption at a given displacement was calculated by integrating the force–displacement curve:

(18) $$EA\left(\delta \right)=\int_{0}^{\delta }F\left(x\right)dx$$

The specific energy absorption was defined as:

(19) SEA=EA/m

where *m* is the mass of the specimen.

The densification displacement, *δ_d_*, was determined using the energy-absorption-efficiency method and was taken as the displacement corresponding to the maximum energy-absorption efficiency.

The corresponding energy-absorption efficiency $\eta \left(\delta \right)$ was defined as:

(20) $$\eta \left(\delta \right)=\frac{EA\left(\delta \right)}{F\left(\delta \right)H_{0}}=\frac{\int_{0}^{\delta }F\left(\delta \right)d\delta }{F\left(\delta \right)H_{0}}$$

The densification displacement was then obtained as:

(21) $$\delta_{d}=\delta \left|\eta \left(\delta \right)=\eta_{\max}\right.$$

The mean crushing force and crushing force efficiency were calculated as:

(22) $$MCF=EA/\delta_{d}$$

(23) $$CFE=MCF/F_{\max}\times 100\%$$

where *MCF* represents the average load-carrying level before densification, and *F*_max_ is the maximum compression force within the same pre-densification displacement range. *CFE* evaluates the stability of the crushing response. A higher *CFE* indicates a smaller difference between the average crushing force and the maximum force, which is desirable for energy absorption applications.

The engineering stress and engineering strain were calculated as:

(24) $$\sigma =F/A_{0}$$

(25) $$\varepsilon =\delta /H_{0}$$

where *F* is the compression force, *δ* is the compression displacement, *A*_0_ is the initial cross-sectional area, and *H*_0_ is the initial specimen height. In this study, *A*_0_ = 900 mm^2^ and *H*_0_ = 30 mm.

#### 2.4.2. Dynamic Impact-Performance Metrics

**Figure 5:**
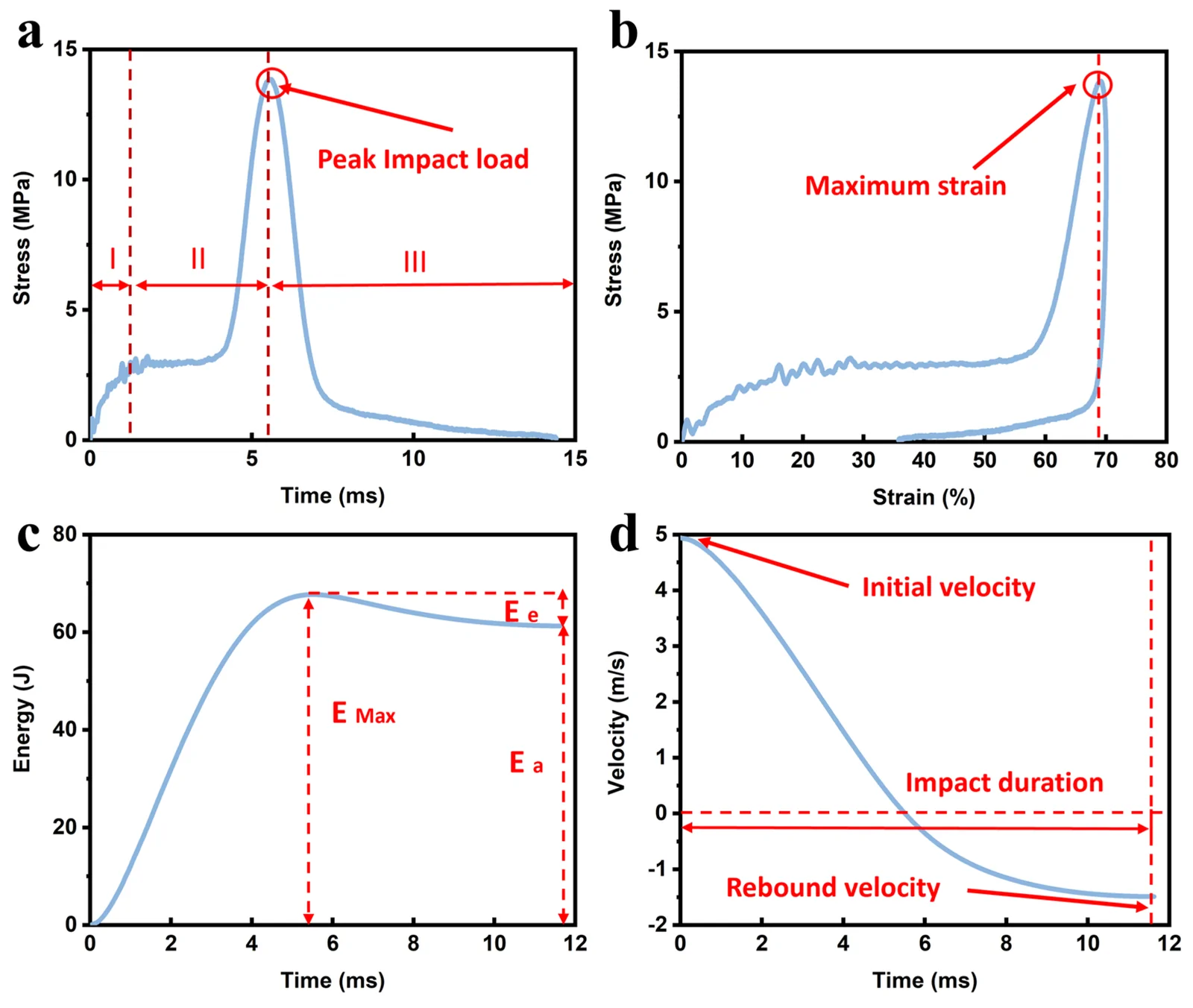
Definition of impact performance metrics: (**a**) peak impact stress from the stress-time curve; (**b**) maximum strain from the stress-strain curve; (**c**) specific energy absorption from the internal energy-time curve; and (**d**) impact duration from the velocity-time curve. The Roman numerals denote the three impact stages: I, initial contact; II, plateau crushing and densification up to maximum compression; and III, rebound from maximum compression to complete separation.

The impact performance of the three TPMS absorbers was evaluated in terms of peak impact stress, maximum strain, specific energy absorption, and impact duration. These metrics characterize the peak loading level, utilization of the available compression stroke, energy uptake per unit mass, and duration of the primary deceleration phase, respectively. Their definitions are illustrated in Figure 5.

The impact response was divided into three stages. Stage I extends from the onset of contact to the onset of plateau crushing and represents the initial loading stage. Stage II comprises plateau crushing and subsequent densification up to the maximum compression, at which the maximum strain and maximum internal-energy uptake are reached. Stage III represents the rebound process from maximum compression until the impactor completely separates from the specimen.

The nominal impact stress $\sigma_{imp}$ was calculated by dividing the total contact force at the upper impact platen by the initial cross-sectional area of the specimen. The peak impact stress $\sigma_{peak}$ was defined as the maximum nominal impact stress during the impact event. A lower peak impact stress indicates a reduced instantaneous loading level at the impact interface, which is desirable for landing-buffer applications.

(26) $$\sigma_{imp}\left(t\right)=\frac{F_{up}\left(t\right)}{A_{0}}$$

(27) $$\sigma_{peak}=\max\left[\sigma_{imp}\left(t\right)\right]$$

The maximum strain $\varepsilon_{\max}$ was calculated by normalizing the maximum relative displacement between the upper and lower platens by the initial specimen height. It represents the largest overall compressive deformation attained during impact and was used to evaluate the utilization of the available deformation stroke. For a given allowable stroke, a larger maximum strain may indicate more effective utilization of the available deformation space, provided that premature densification is avoided.

(28) $$\varepsilon_{\max}=\frac{\delta_{\max}}{H_{0}}$$

The energy uptake of the TPMS absorber under impact loading was evaluated from the increase in its internal energy $\Delta E_{int}$, which differs from the energy absorption calculation used in the quasi-static compression tests in Equation (19).

(29) $$SEA_{imp}=\frac{\Delta E_{int}}{m_{s}}$$

The contact duration was defined as the time interval between the onset of platen–specimen contact and the final loss of contact, as determined from the contact–force history. This instant corresponds to the maximum absorbed energy of the absorber and marks the end of the primary deceleration stage.

For a given impact energy and allowable compression stroke, a longer impact duration generally indicates a more gradual deceleration process; however, it should be evaluated together with the peak impact stress, maximum strain, and residual or rebound response.

## 3. Quasi-Static Compression Screening of Uniform TPMS Structures

### 3.1. Voxel-Size Convergence and Computational Efficiency

A voxel-size convergence analysis was conducted to determine an appropriate voxel resolution for the subsequent simulations. The single P20 unit cell was selected as the representative model for this analysis because it was used as one of the baseline topologies in the screening stage. Four voxel resolutions were considered, corresponding to 15, 20, 30, and 40 divisions per unit cell. Given a unit cell size of 10 mm, these resolutions correspond to voxel sizes of approximately 0.66 mm, 0.50 mm, 0.33 mm, and 0.25 mm, respectively.

The geometric discretization error was quantified by comparing the relative density of the voxelized model with the prescribed target relative density.

(30) $$\rho_{er}=\left|\rho_{vox}-\rho_{tar}\right|\times 100\%$$

where $\rho_{vox}$ is the actual relative density after voxelization and $\rho_{tar}$ is the prescribed target relative density of the original TPMS model. The number of elements and solid volume were also recorded to assess the geometric accuracy and computational cost associated with each voxel resolution.

As summarized in Table 2, increasing the voxel resolution progressively improved the geometric representation of the single 10 mm P20 unit cell. Table 2 reports the voxelized solid volume of this unit cell, while the full 30 mm × 30 mm × 30 mm specimen used in the subsequent analyses was constructed by arranging 3 × 3 × 3 identical unit cells, so its total solid volume is 27 times the unit-cell volume. The relative density increased from 19.200% at 15 voxels per unit-cell direction to 19.881% at 40 voxels per unit-cell direction, while the corresponding density error decreased from 4.000% to 0.595%. This improvement was accompanied by a substantial increase in the number of elements, from 648 to 12,724.

**Figure 6:**
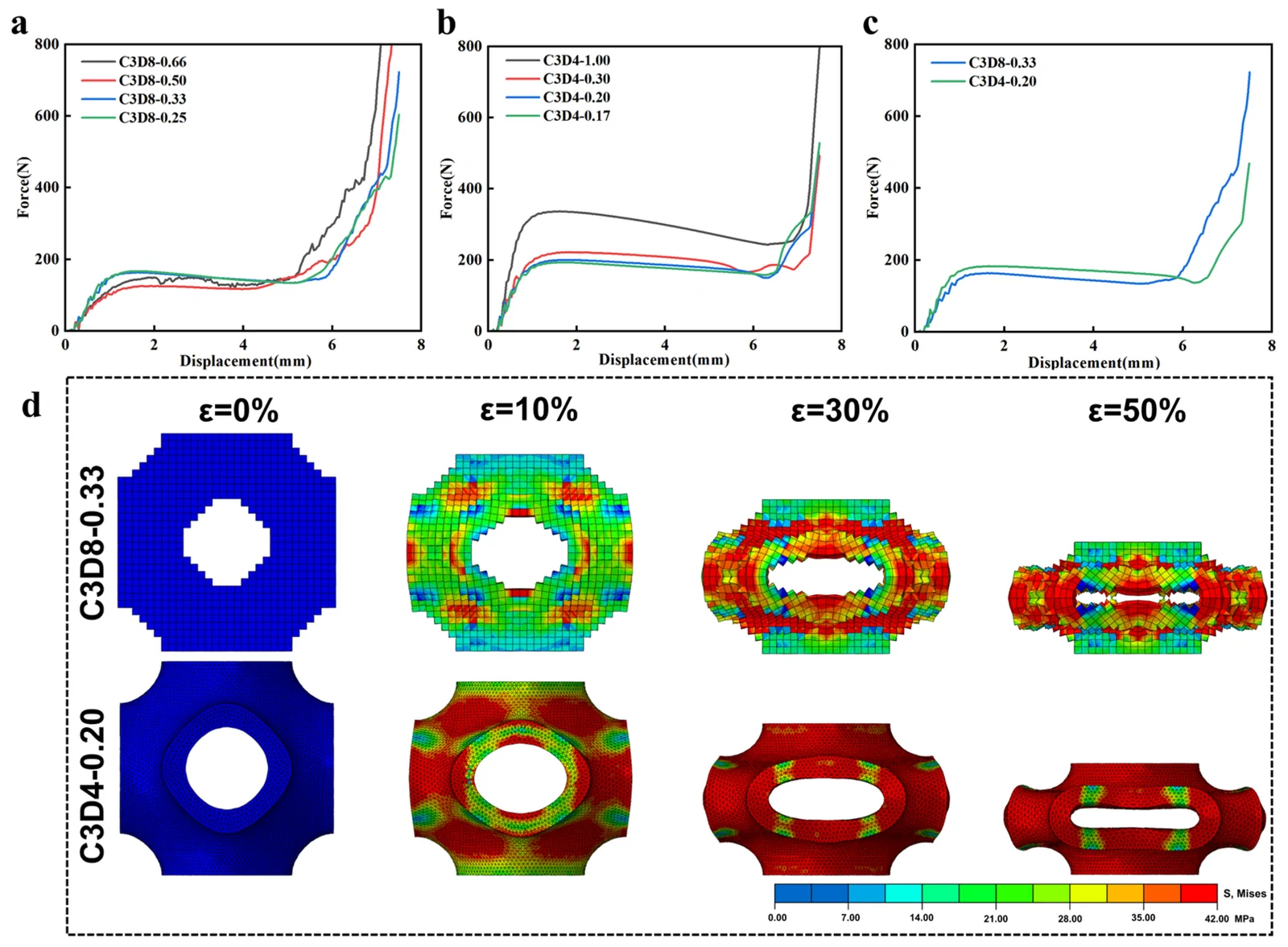
Mesh convergence and comparison between voxelized and tetrahedral models: (**a**) voxel-size convergence analysis of the C3D8R voxelized model; (**b**) mesh convergence analysis of the C3D4 tetrahedral model; (**c**) force–displacement comparison between the 0.33 mm voxelized mesh and the 0.2 mm tetrahedral mesh; and (**d**) stress contour comparison of the P20 structure between the 0.33 mm voxelized mesh and the 0.2 mm tetrahedral mesh.

The mechanical convergence behavior of the voxelized model was evaluated using the initial stiffness and force–displacement curve of the P20 specimen. As shown in Figure 6a, refinement beyond 30 voxels per unit-cell direction resulted in only minor changes in the global force–displacement response. In particular, the differences in initial stiffness and peak compression force between the 0.333 and 0.250 mm models were both below 5%. Therefore, a resolution of 30 voxels per unit-cell direction, corresponding to a voxel size of 0.333 mm, was selected as an appropriate balance between geometric accuracy, mechanical convergence, and computational cost.

**Figure A1:**
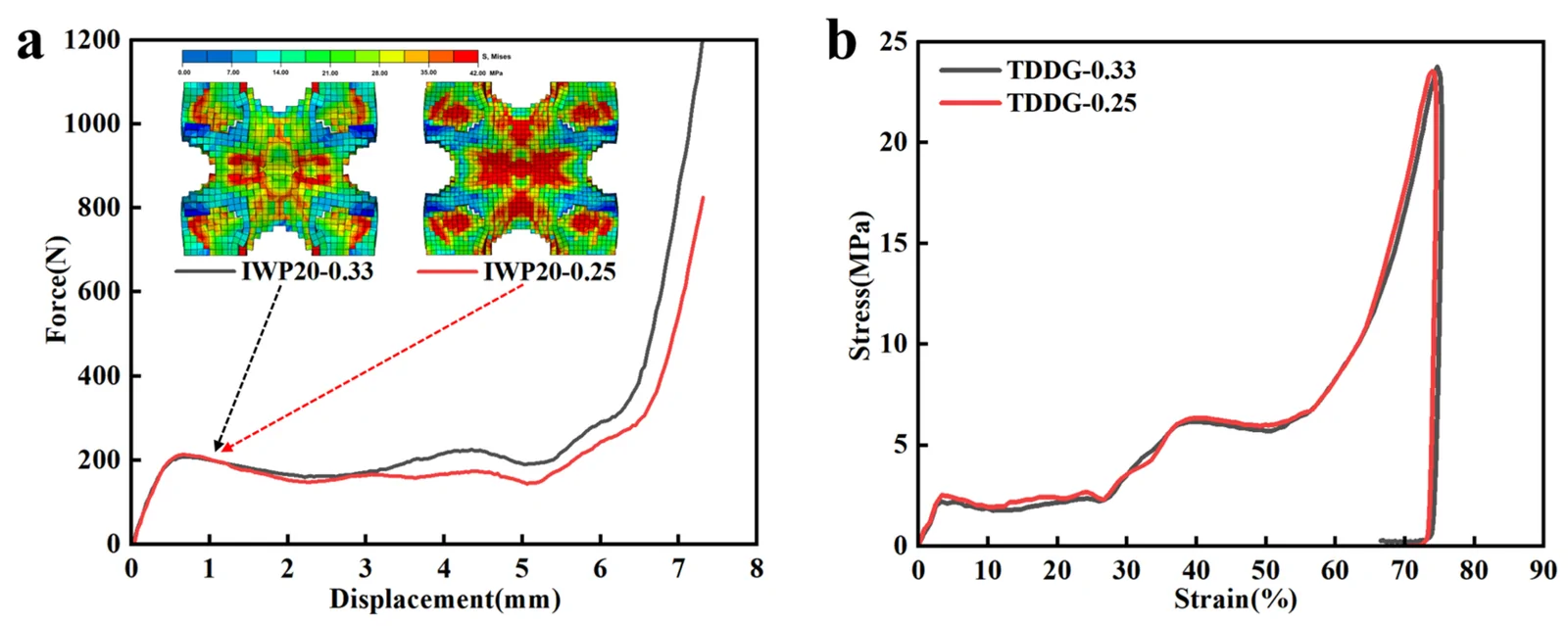
Additional voxel-size convergence comparisons: (**a**) quasi-static force–displacement responses of IWP20 obtained using voxel sizes of 0.33 and 0.25 mm; and (**b**) nominal stress–strain responses of TDDG-IWP20-P40 obtained using voxel sizes of 0.33 and 0.25 mm under the 125 J impact condition.

For comparison, conventional tetrahedral models composed of C3D4 elements were generated with characteristic element sizes of 1.0, 0.3, 0.2, and 0.17 mm. Based on the convergence results presented in Figure 6b, the tetrahedral model with an element size of 0.2 mm was selected as the reference model. Figure 6c shows that the 0.333 mm voxelized C3D8R model and the 0.2 mm tetrahedral C3D4 model produced comparable global force–displacement responses, indicating that the voxel-based discretization provided sufficient accuracy for predicting the global compression response of the TPMS structure. Additional voxel-size verification was performed for the thinner IWP20 structure under quasi-static compression and for the graded TDDG-IWP20-P40 structure under impact loading. The comparative response curves and quantitative convergence results for voxel sizes of 0.33 and 0.25 mm are provided in Appendix A (Figure A1 and Table A1). The close agreement between the two resolutions further confirms that the selected 0.33 mm voxel size is sufficient for the global mechanical responses considered in this study.

The computational efficiency of the two discretization strategies was further compared under the same hardware and solver settings. The wall-clock time was 4222 s for the 0.2 mm tetrahedral model and 497 s for the 0.33 mm voxelized model. Therefore, the voxelized model reduced the computational time by approximately 88.2% while maintaining a comparable mechanical response. This result demonstrates that the voxelization method offers a computationally efficient alternative to conventional tetrahedral meshing for the TPMS compression analyses in this work.

### 3.2. Experimental Validation of the Numerical Model

Following the voxel-size convergence and computational-efficiency analyses presented in Section 3.1, the predictive capability of the selected voxel-based C3D8R model was first assessed using the P20 3 × 3 × 3 array. Therefore, the model size is 30 mm × 30 mm × 30 mm. The numerical force–displacement response was compared with the corresponding quasi-static compression result. To further examine the applicability of the modeling approach across different topologies and relative densities, the validation was subsequently extended to all nine P, G, and IWP configurations. The numerical and experimental results were compared in terms of both deformation mode and global force–displacement response.

**Figure 7:**
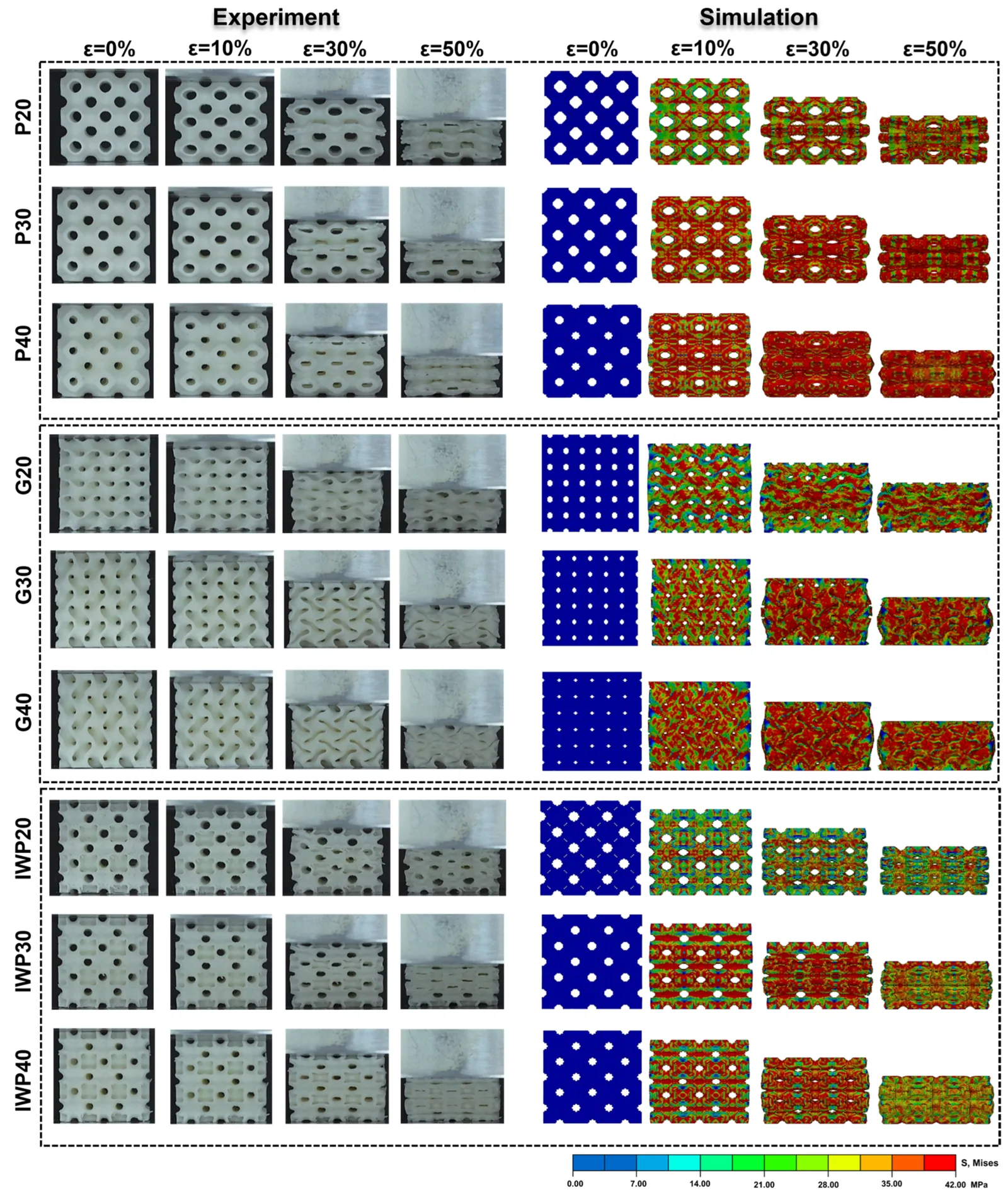
Comparison of experimental and numerical deformation modes of P, G, IWP structures at compression strains of 0%, 10%, 30%, and 50%.

Figure 7 compares the experimentally observed and numerically predicted deformation modes of the P, G, and IWP structures at nominal compressive strains of 0%, 10%, 30%, and 50%. The simulations reproduced the principal deformation characteristics observed experimentally, including the initiation of structural collapse, progressive compaction, and the transition toward densification at large strains. Although minor differences were observed in the locations and sequence of localized collapse, the predicted global deformation patterns were generally consistent with the experimental observations.

**Figure 8:**
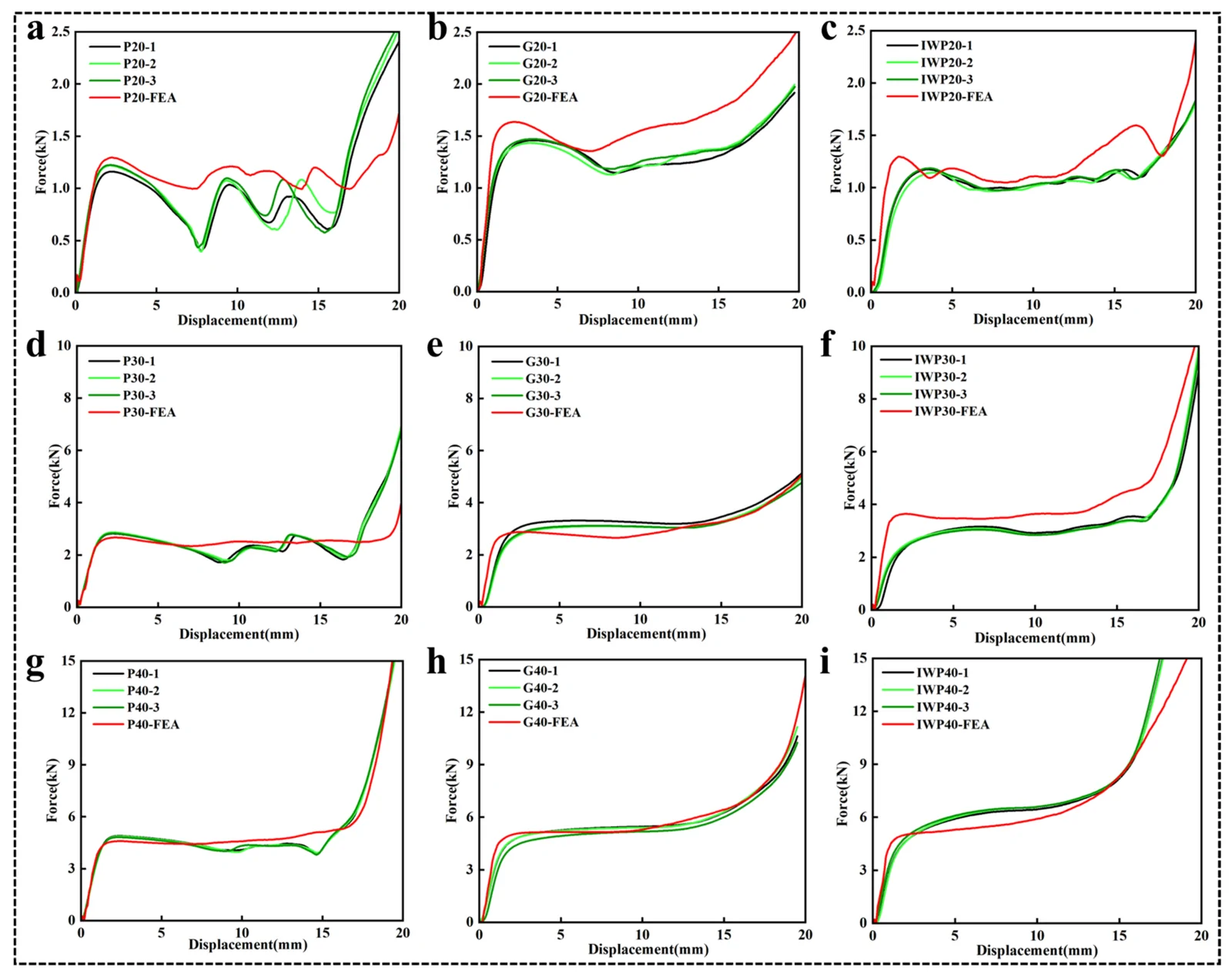
Experimental and simulation force–displacement curves of the P, G, and IWP TPMS structures. (**a**–**c**) 20% relative density; (**d**–**f**) 30% relative density; (**g**–**i**) 40% relative density.

The experimental and numerical force–displacement responses of the nine TPMS configurations are presented in Figure 8. For each configuration, the results from three independently tested specimens are shown together with the corresponding numerical prediction. The experimental curves demonstrate the repeatability and specimen-to-specimen variability of the fabricated structures, whereas the numerical curves represent the responses predicted using the idealized voxel-based models. In general, the simulations reproduced the initial stiffness, crushing-force level, and transition toward densification. The numerical models also captured the effects of topology and relative density on the global compression response.

Some local force fluctuations observed experimentally were not fully reproduced by the numerical models. These discrepancies can primarily be attributed to printing-induced geometric imperfections, layerwise material anisotropy, and local damage or fracture. These effects were not explicitly represented in the idealized finite element models, in which the printed material was treated as homogeneous and isotropic. Moreover, small manufacturing imperfections may have influenced the initiation and location of localized collapse in the experiments, whereas collapse in the numerical models was governed by the nominal geometry and numerical perturbations.

Despite these local discrepancies, the voxel-based models captured the principal deformation and load-bearing characteristics of the fabricated TPMS specimens. The overall agreement between the experimental and numerical deformation modes and force–displacement responses demonstrates that the selected C3D8R voxel-based framework provides sufficient accuracy for predicting the global quasi-static compression behavior considered in this study. The experimentally measured compression responses were subsequently used to obtain the stress–strain curves and calculate the energy-absorption metrics according to the definitions provided in Section 2.4.1, while the numerical responses were used only for model validation.

### 3.3. Density-Dependent Compression and Energy-Absorption Performance

**Figure 9:**
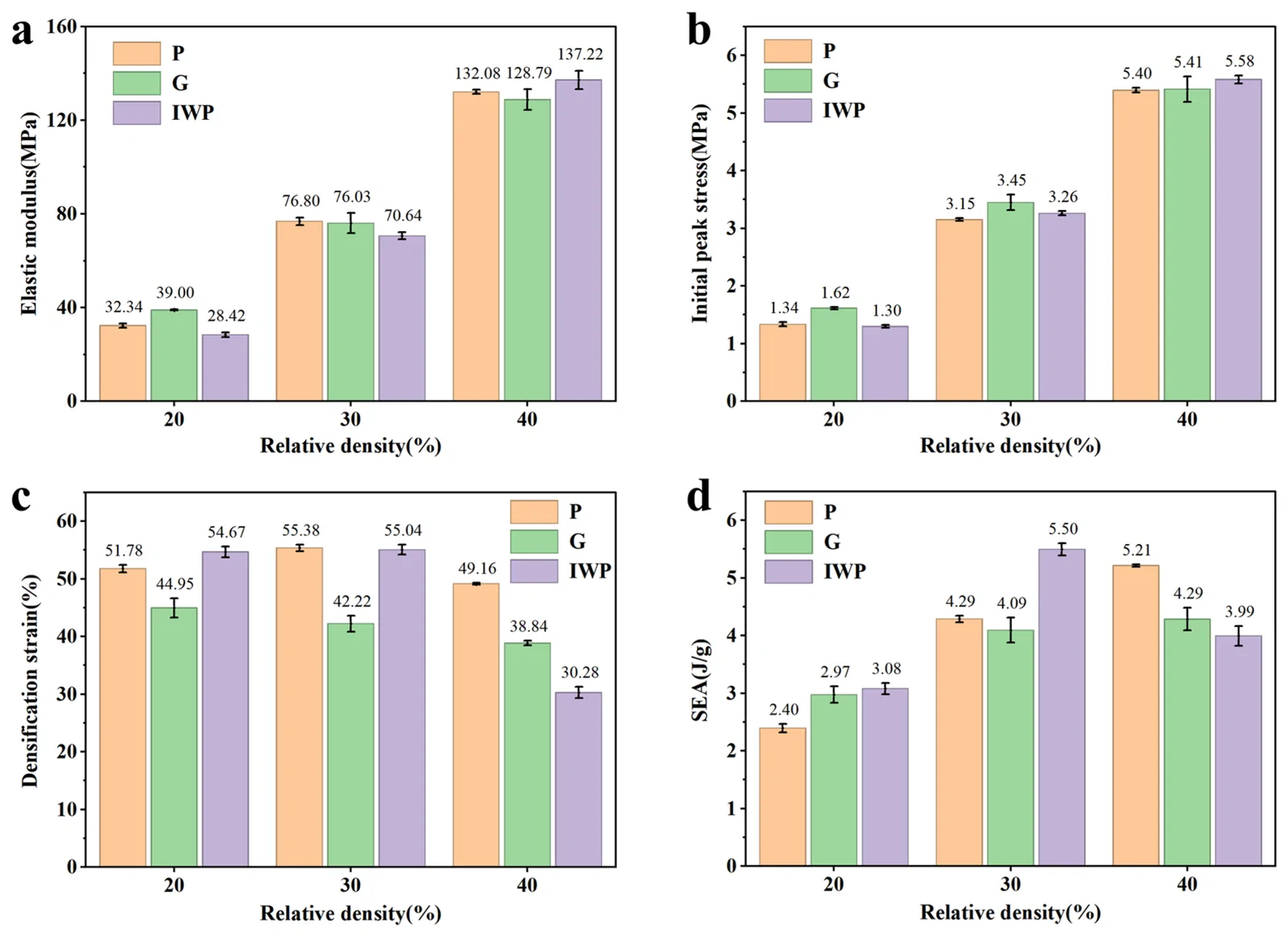
Comparison of the quasi-static performance metrics of the P, G, and IWP structures at different relative densities: (**a**) elastic modulus; (**b**) initial peak stress; (**c**) densification strain; and (**d**) specific energy absorption.

The quasi-static performance metrics extracted from the experimental compression responses are summarized in Table 3, and the density-dependent variations in the principal metrics are further compared in Figure 9. For all three topologies, increasing the relative density resulted in higher effective elastic moduli and initial peak stresses, indicating enhanced stiffness and load-bearing capacity. However, the variations in densification strain, specific energy absorption, and crushing force efficiency were strongly dependent on topology.

At a relative density of 20%, IWP20 exhibited favorable compression and energy-absorption characteristics. Its initial peak stress was 1.299 MPa, which was comparable to that of P20 (1.336 MPa) and lower than that of G20 (1.617 MPa). Meanwhile, IWP20 achieved the highest SEA among the three low-density configurations, reaching 3.079 J/g, compared with 2.395 J/g for P20 and 2.975 J/g for G20. It also exhibited the highest densification strain (54.67%). Its CFE of 85.33% was essentially comparable to that of G20 (85.31%) and higher than that of P20 (71.92%). These results indicate that IWP20 combined a low initial loading level with a relatively long effective compression stroke and stable crushing response.

At a relative density of 30%, IWP30 provided the highest SEA of 5.496 J/g, compared with 4.287 J/g for P30 and 4.091 J/g for G30. P30 and IWP30 exhibited essentially comparable densification strains of 55.38% and 55.04%, respectively, while both values were higher than that of G30 (42.22%). P30 exhibited the lowest initial peak stress of 3.151 MPa, whereas G30 achieved the highest CFE of 90.96% among the three 30% relative-density configurations. Thus, IWP30 showed the greatest mass-specific energy absorption, P30 combined a lower initial peak stress with a comparable effective compression stroke, and G30 exhibited the most uniform crushing response. The 30% relative-density level was subsequently adopted as the average-density reference for comparing the uniform and topology–density dual-gradient absorbers.

At a relative density of 40%, the differences in densification behavior had a dominant influence on the energy-absorption performance. IWP40 exhibited the highest effective elastic modulus of 137.217 MPa, whereas G40 achieved the highest CFE of 87.76% among the three 40% relative-density configurations. However, the densification strain of IWP40 decreased to 30.28%, indicating a substantially shortened effective compression stroke. Its SEA consequently decreased from 5.496 J/g at 30% relative density to 3.994 J/g at 40%. G40 showed a similar reduction in densification strain, reaching 38.84%, although its SEA increased moderately to 4.286 J/g. In comparison, P40 retained a relatively high densification strain of 49.16% and achieved an SEA of 5.213 J/g, the highest value among the three 40% relative-density configurations. Moreover, its initial peak stress of 5.396 MPa was lower than those of G40 and IWP40. Therefore, P40 provided a more favorable balance among initial loading level, effective compression stroke, and mass-specific energy absorption at high relative density.

The cross-density comparison further demonstrates that increasing relative density did not lead to a uniform improvement in energy-absorption performance across the three topologies. For the P topology, SEA increased continuously from 2.395 to 5.213 J/g as the relative density increased from 20% to 40%, while the densification strain remained within a relatively narrow range of 49.16–55.38%. For the G topology, SEA increased moderately with relative density, but the densification strain progressively decreased from 44.95% to 38.84%. The IWP topology exhibited the strongest density sensitivity: its SEA reached a maximum at 30% relative density and subsequently decreased at 40%, accompanied by a pronounced reduction in densification strain from 55.04% to 30.28%.

Overall, no single topology exhibited superior performance across all relative densities and evaluation metrics. IWP showed favorable low-density characteristics, particularly in terms of its low initial peak stress, high densification strain, and SEA, whereas P retained a longer effective compression stroke and higher SEA at elevated relative density. Although the G structures exhibited relatively high CFE, particularly at relative densities of 30% and 40%, they did not provide the same combination of densification strain and SEA as IWP20 at low density or P40 at high density. These complementary topology- and density-dependent characteristics provide the basis for selecting the impact-facing region and support-facing region constituents of the topology–density dual-gradient TPMS absorber in Section 3.4.

### 3.4. Topology Selection for the Topology–Density Dual-Gradient TPMS Absorber

The density-dependent results presented in Section 3.3 demonstrate that the preferred TPMS topology depends on both relative density and the functional requirements of the corresponding region. Accordingly, the constituent configurations of the topology–density dual-gradient (TDDG) TPMS absorber were selected using region-specific criteria rather than a single performance metric. The impact-facing region was required to initiate progressive deformation at a relatively low loading level, maintain stable crushing, and provide a sufficiently long effective compression stroke. In contrast, the support-facing region was required to provide sufficient structural support and energy-absorption capacity while avoiding premature densification.

Among the configurations with a relative density of 20%, IWP20 provided the most favorable combination of performance metrics. It exhibited the lowest initial peak stress, the highest SEA, and the largest densification strain among the three low-density structures. Its relatively high CFE also indicated efficient utilization of the peak load during progressive compression. Collectively, these characteristics suggest that IWP20 can initiate deformation at a relatively low stress level while maintaining an extended effective crushing stroke. IWP20 was therefore selected as the low-density constituent of the impact-facing region.

At a relative density of 40%, P40 exhibited the most balanced performance for the support-facing region. Although it did not possess the highest effective elastic modulus among the high-density configurations, it achieved the highest SEA of all nine uniform structures and retained a substantially larger densification strain than G40 and IWP40. In addition, its initial peak stress was lower than those of the other two high-density configurations. Thus, P40 provided sufficient high-density structural support without the pronounced premature densification observed for G40 and IWP40. It was therefore selected as the high-density constituent of the support-facing region.

The G topology was retained during the screening stage to evaluate the topology dependence of the quasi-static response. However, the G configurations did not exhibit a distinct functional advantage over IWP20 for low-density progressive crushing or over P40 for high-density support and energy absorption. Hence, G was not selected as either end constituent of the TDDG design.

Based on these complementary characteristics, the subsequent TDDG absorber was constructed using IWP20 at the impact-facing end and P40 at the support-facing end. A sigmoid-based interpolation scheme was introduced between the two end configurations to generate coordinated and smooth spatial variations in topology and relative density, as detailed in Section 4.1. The present quasi-static screening establishes the constituent-selection basis for the TDDG design; however, its dynamic load-mitigation and energy-absorption performance must be assessed independently under impact loading. These dynamic responses are investigated in Section 4.

## 4. Impact Performance of the Topology–Density Dual-Gradient TPMS Absorber

### 4.1. Design of the IWP-P Topology–Density Dual-Gradient TPMS Absorber

Based on the constituent-screening results presented in Section 3, an IWP-P topology–density dual-gradient (TDDG) TPMS absorber was developed by exploiting the complementary density-dependent characteristics of the IWP and P topologies. IWP20 and P40 were assigned to the impact-facing and support-facing ends, respectively. Unlike a uniform TPMS structure, the proposed design enables the local topology, relative density, stiffness, and collapse resistance to vary continuously along the impact direction.

The low-density IWP region at the impact-facing end was designed to initiate deformation at a relatively low loading level, thereby mitigating the initial force peak and promoting progressive crushing during the early stage of impact. In contrast, the high-density P region at the support-facing end was intended to provide increased resistance and maintain an extended effective crushing stroke during the later stage of deformation. This impact-to-support arrangement was designed to balance initial load mitigation, progressive collapse, and energy-absorption capacity. Nevertheless, because the constituent configurations were selected from quasi-static screening, the effectiveness of this design under dynamic loading was evaluated independently in the subsequent sections.

To avoid an abrupt interface between the two constituent configurations, a sigmoid-based interpolation function was introduced to coordinate the spatial variations in topology and relative density. The normalized coordinate along the specimen height was defined as

(31) $$\xi =\frac{z}{H},\;0\leq \xi \leq 1$$

where *z* is the coordinate measured from the impact-facing end and *H* is the total specimen height. The unnormalized sigmoid function was expressed as

(32) $$S\left(\xi \right)=\frac{1}{1+\exp\left[-k\left(\xi -\xi_{0}\right)\right]}$$

where $\xi_{0}$ specifies the center of the transition and *k* controls its sharpness. To ensure that the interpolation weight varied exactly from 0 at the impact-facing end to 1 at the support-facing end, the sigmoid function was normalized as

(33) $$w\left(\xi \right)=\frac{S\left(\xi \right)-S\left(0\right)}{S\left(1\right)-S\left(0\right)}$$

In the present design, the transition center and sharpness parameter were set to $\xi_{0}=0.5$ and *k* = 1, respectively. Thus, the transition was centered at the mid-height of the specimen and distributed smoothly along the impact direction.

**Figure 10:**
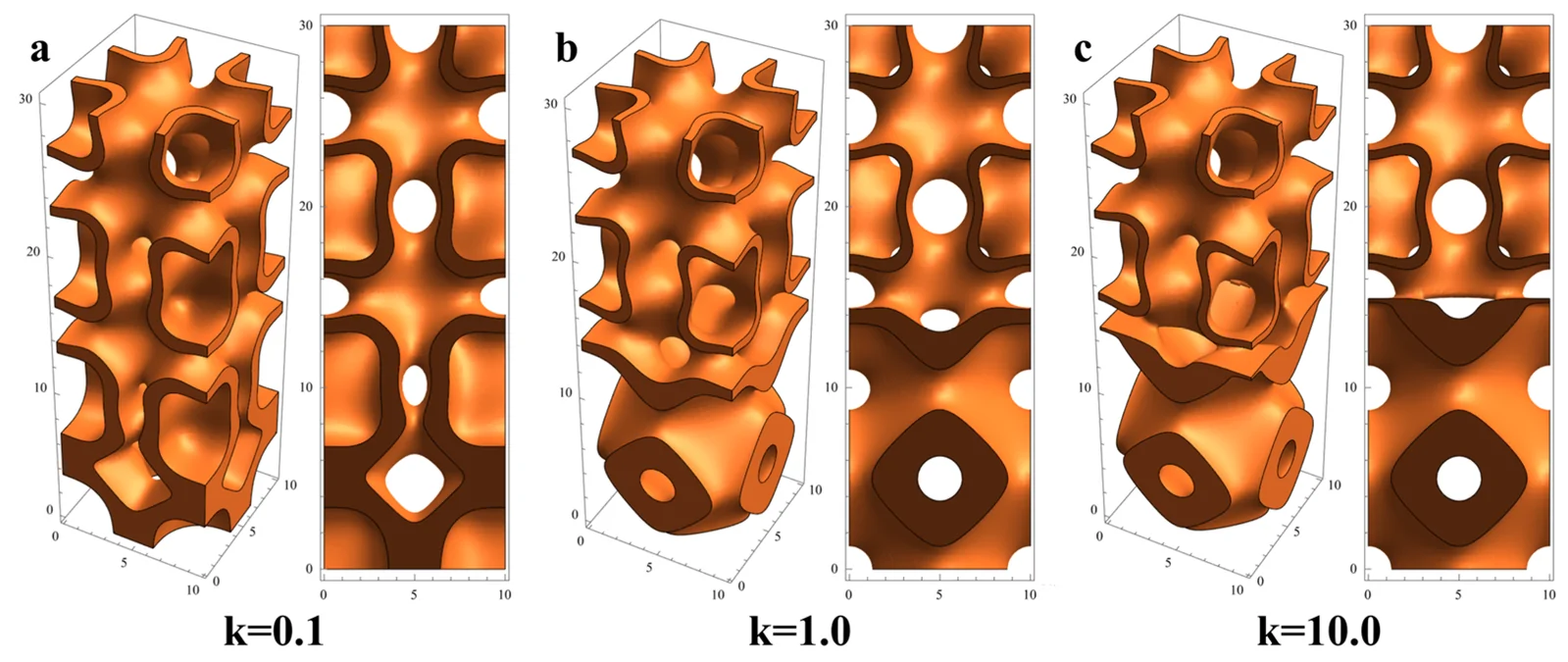
Local transition morphology of the TDDG structure generated using different sigmoid interpolation parameters: (**a**) *k* = 0.1, (**b**) *k* = 1, and (**c**) *k* = 10. A smaller *k* produces a smoother and broader transition region, whereas a larger *k* leads to a sharper and more localized topology transition.

To further illustrate the local transition morphology, Figure 10 compares the TDDG transition regions generated using different sigmoid sharpness parameters. A smaller *k* produces a smoother and broader transition region, whereas a larger *k* leads to a sharper and more localized topology transition. The value *k* = 1 used in this study provides a gradual IWP-to-P transition without introducing an abrupt interface or isolated thin features.

The prescribed local relative density was determined using the same interpolation weight:

(34) $$\rho \left(\xi \right)=\left[1-w\left(\xi \right)\right]\rho_{IWP}+w\left(\xi \right)\rho_{P}$$

where $\rho_{IWP}=0.20$ and $\rho_{P}=0.40$. Consequently, the prescribed relative density increased continuously from 20% at the impact-facing end to 40% at the support-facing end, while maintaining an average relative density of approximately 30% for the symmetric transition adopted in this study.

The topology transition was generated by interpolating the implicit functions of the IWP and P surfaces:

(35) $$\Phi \left(x,y,z\right)=\left[1-w\left(\xi \right)\right]\Phi_{IWP}\left(x,y,z\right)+w\left(\xi \right)\Phi_{P}\left(x,y,z\right)$$

where $\Phi_{IWP}$ and $\Phi_{P}$ denote the implicit functions of the IWP and P topologies, respectively. The local level-set or thickness parameter was adjusted according to the prescribed density distribution $\rho \left(\xi \right)$, thereby coordinating the topology transition with the relative-density gradient. At ξ=0, the interpolated field reduces to the IWP topology with a target relative density of 20%, whereas at ξ=1, it reduces to the P topology with a target relative density of 40%.

The resulting structure was designated as TDDG-IWP20-P40, indicating an IWP-to-P topology transition accompanied by a relative-density increase from 20% to 40% along the impact direction. The dynamic impact performance of this TDDG configuration was subsequently compared with that of the corresponding uniform reference structure under identical mass, boundary, and loading conditions.

### 4.2. Impact Simulation Cases and Numerical Setup

**Figure 11:**
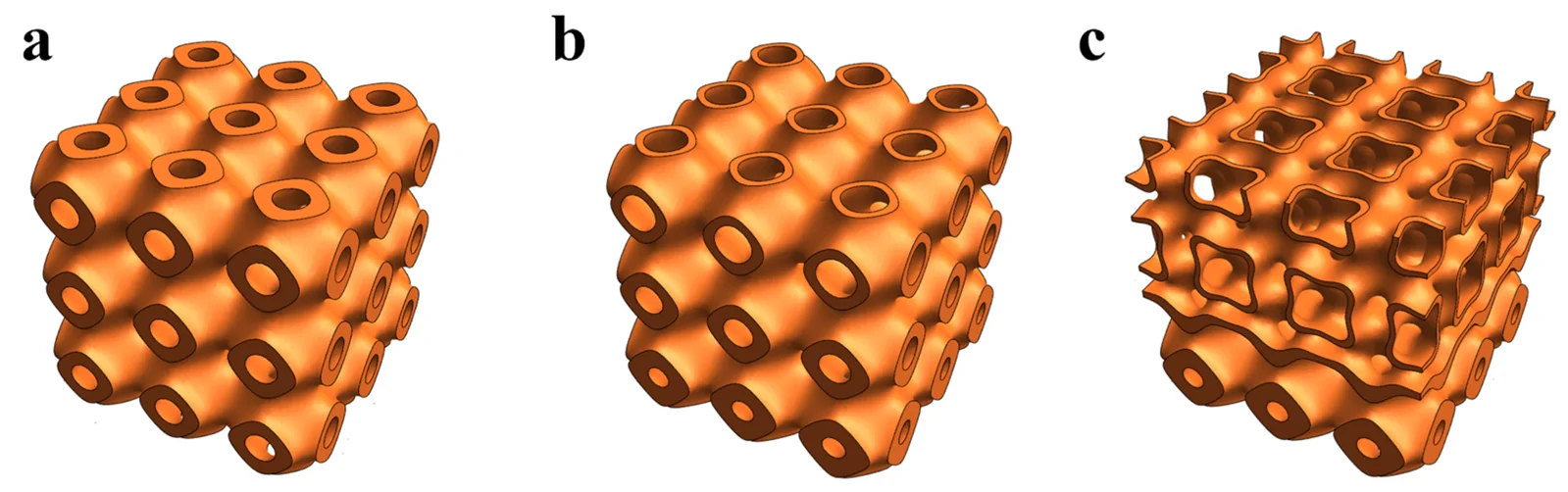
Impact simulation models of the three TPMS energy absorbers: (**a**) U-P30, Uniform P structure with 30% relative density; (**b**) DG-P20-40, density-graded P structure with a 20–40% density gradient; and (**c**) TDDG-IWP20-P40, topology–density dual-gradient structure transitioning from IWP20 at the impact-facing end to P40 at the support-facing end.

Three impact simulation cases were established to distinguish the effects of density grading and topology–density coupling, as illustrated in Figure 11. The uniform P structure with a relative density of 30% (U-P30) was adopted as the baseline configuration. The density-graded P structure, in which the relative density increased from 20% at the impact-facing end to 40% at the support-facing end (DG-P20-P40), was used to evaluate the effect of density grading within a single topology. The proposed IWP-P topology–density dual-gradient structure (TDDG-IWP20-P40) incorporated simultaneous variations in topology and relative density. All three configurations had identical external dimensions and a nominal average relative density of 30%, allowing their impact responses to be compared under equivalent geometric and mass-related conditions.

The impact simulations were performed using the Dynamic, Explicit procedure in Abaqus/Explicit. The TPMS specimens were discretized using the voxel-based C3D8R models described in Section 2.2, with the voxel size determined through the convergence study in Section 3.1. Each specimen had external dimensions of 30 mm × 30 mm × 30 mm. The same element formulation, material model, and general contact treatment were employed for all three configurations to ensure a consistent comparison.

The specimen was positioned between two analytically rigid plates. The lower plate was fully constrained, whereas the upper plate was allowed to translate only along the axial impact direction, with all other translational and rotational degrees of freedom constrained. A concentrated mass of 10 kg was assigned to the reference point of the upper plate. An initial velocity of 5 m/s was prescribed along the compression direction, corresponding to an initial kinetic energy of 125 J.

To evaluate the robustness of the peak-stress mitigation under different impact conditions, two additional sets of simulations were conducted. A 5 kg impactor was assigned initial velocities of 5 and 8 m/s, corresponding to initial kinetic energies of 62.5 and 160 J, respectively. Together with the reference condition of 10 kg and 5 m/s (125 J), these cases covered lower and higher initial impact-energy levels. Under each condition, U-P30, DG-P20-P40, and TDDG-IWP20-P40 were analyzed using identical material models, boundary conditions, contact definitions, and numerical settings.

A total analysis time of 10 ms was adopted for each impact simulation. This duration was sufficient to capture the initial contact, progressive collapse, substantial energy transfer, and the onset of either specimen densification or impactor rebound. The contact definitions were consistent across all impact cases. Surface-to-surface contact was defined between the TPMS specimen and the two rigid plates, and self-contact was introduced within the TPMS structure to account for internal contact between opposing surfaces during large deformation and densification. Hard contact was used in the normal direction to prevent surface penetration. Tangential contact was described using the penalty formulation, with friction coefficients of 0.2 between the specimen and rigid plates and 0.3 for self-contact within the TPMS specimen.

**Figure A2:**
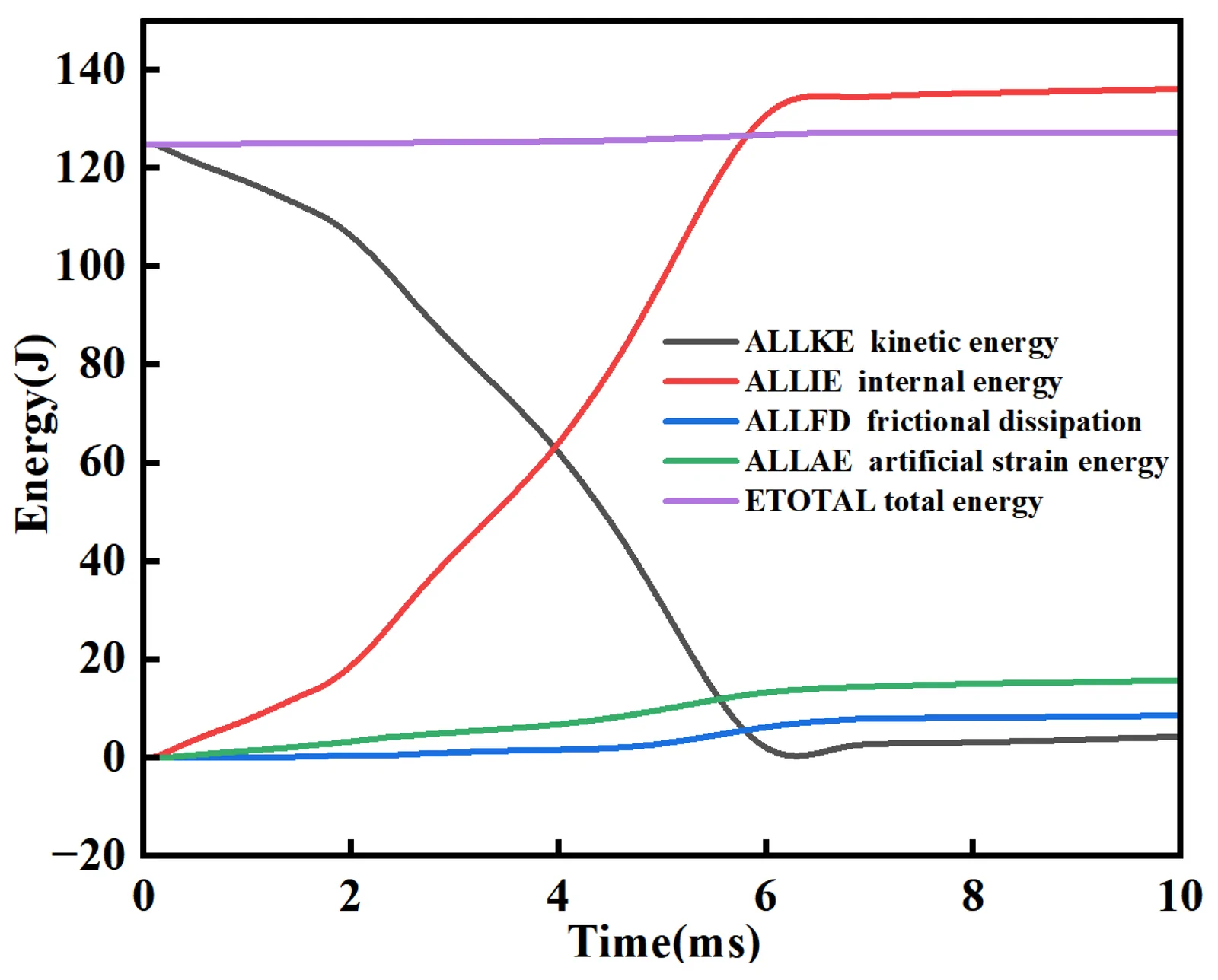
Numerical energy balance of the TDDG-IWP20-P40 model under the 125 J impact condition, including kinetic energy (ALLKE), internal energy (ALLIE), frictional dissipation energy (ALLFD), artificial strain energy (ALLAE), and total energy (ETOTAL).

To assess the numerical stability of the explicit impact simulations, the histories of kinetic energy (ALLKE), internal energy (ALLIE), total energy (ETOTAL), artificial strain energy (ALLAE), and frictional dissipation energy (ALLFD) were examined for the TDDG-IWP20-P40 model. ETOTAL remained nearly constant, with a maximum deviation of 1.75% from its initial value. At the end of the simulation, ALLAE was approximately 11% of ALLIE. Although the artificial energy was not negligible during severe final-stage crushing and self-contact, it remained substantially lower than the internal energy and did not dominate the global response. The complete energy histories are provided in Appendix A.2 (Figure A2).

### 4.3. Impact Energy-Absorption Performance

**Figure 12:**
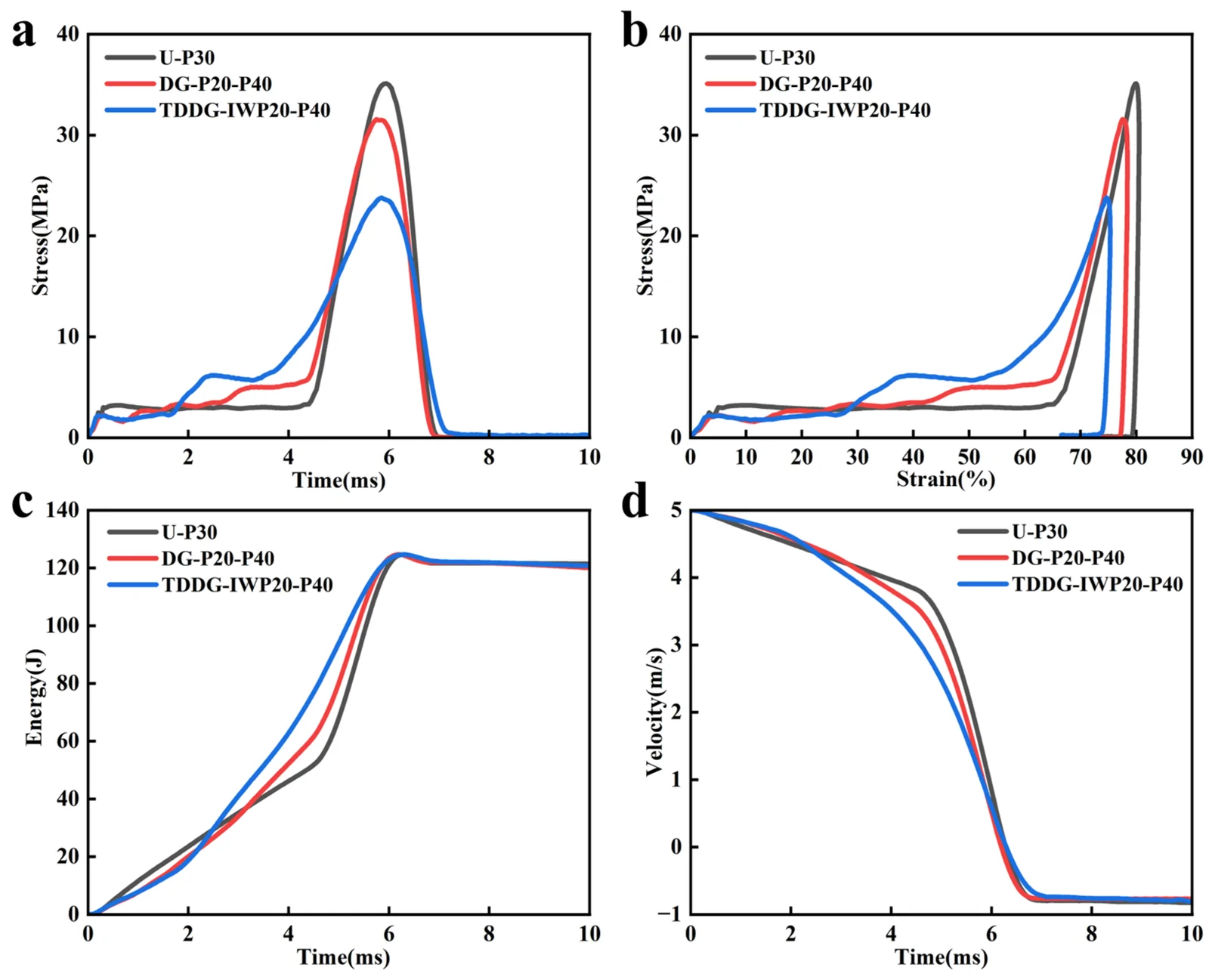
Dynamic impact responses of the three TPMS energy absorbers: (**a**) impact stress–time histories; (**b**) impact stress–strain responses; (**c**) internal energy–time histories; and (**d**) impactor velocity–time histories.

The dynamic responses of U-P30, DG-P20-P40, and TDDG-IWP20-P40 under the identical impact condition are compared in Figure 12. The impact stress–time, stress–strain, internal energy–time, and impactor velocity–time histories were examined to evaluate the influence of density grading and coupled topology–density grading. The corresponding impact-performance metrics are summarized in Table 4.

All three specimens were subjected to an initial impact energy of 125 J and had the same nominal average relative density. Consequently, their SEA values and maximum compressive strains remained within relatively narrow ranges. The SEA values varied from 12.151 to 12.287 J/g, corresponding to a maximum difference of approximately 1.1% among the three configurations. These results indicate that the principal advantage of the graded designs was not an increase in the final amount of absorbed energy, but a redistribution of energy dissipation over the impact duration and compression stroke.

The uniform U-P30 structure exhibited a rapid increase in impact stress as densification developed, producing a peak impact stress of 35.105 MPa. Introducing a density gradient within the P topology reduced the peak stress of DG-P20-P40 to 31.544 MPa, representing a reduction of 10.1% relative to U-P30. The TDDG-IWP20-P40 structure further reduced the peak stress to 23.786 MPa. This value was 32.2% lower than that of U-P30 and 24.6% lower than that of DG-P20-P40. Thus, under the adopted numerical assumptions, density grading alone provided a moderate load-mitigation effect, whereas introducing topology variation on top of the same density-gradient profile produced a further reduction in the global impact-stress peak.

The stress–strain responses further demonstrate the influence of the graded architectures on the progression toward densification. U-P30 reached a maximum compressive strain of 79.95%, compared with 77.86% for DG-P20-P40 and 75.02% for TDDG-IWP20-P40. Despite reaching the lowest maximum strain, TDDG-IWP20-P40 absorbed 12.223 J/g, which was only 0.5% lower than the SEA of U-P30. This indicates that the TDDG configuration absorbed a comparable amount of energy over a smaller compression, thereby reducing the need for severe final-stage compaction.

The internal energy histories provide further insight into this behavior. Although the three structures ultimately accumulated similar internal energies, TDDG-IWP20-P40 exhibited a more progressive increase in internal energy during the intermediate stage of impact. A greater proportion of the initial kinetic energy was therefore dissipated before the onset of severe global densification. In contrast, U-P30 retained more energy to be dissipated during the late compression stage, resulting in a sharper increase in impact stress. DG-P20-P40 showed an intermediate response, confirming that density grading improved the temporal distribution of energy absorption but was less effective than the coupled topology–density design.

Consistent with the internal energy histories, the impactor velocity decreased more gradually for the graded configurations. U-P30 exhibited the shortest impact duration of 7.05 ms, whereas the impact durations of DG-P20-P40 and TDDG-IWP20-P40 increased to 7.25 and 7.60 ms, respectively. The TDDG structure therefore extended the deceleration period by 7.8% relative to U-P30 and by 4.8% relative to DG-P20-P40. The longer impact duration, together with the reduced peak stress, indicates that TDDG-IWP20-P40 moderated the transfer of momentum and produced a smoother deceleration process.

The three structures absorbed comparable amounts of impact energy, but differed substantially in their load-transfer and energy-dissipation histories. U-P30 concentrated a larger portion of energy dissipation in the final densification stage, leading to the highest impact-stress peak. Density grading partially alleviated this behavior, whereas the TDDG architecture more effectively distributed crushing and energy absorption over time. As a result, TDDG-IWP20-P40 achieved the lowest peak stress, the longest impact duration, and a comparable SEA without requiring the largest compression strain. The deformation sequence responsible for this load-mitigation mechanism is examined in Section 4.4.

**Figure 13:**
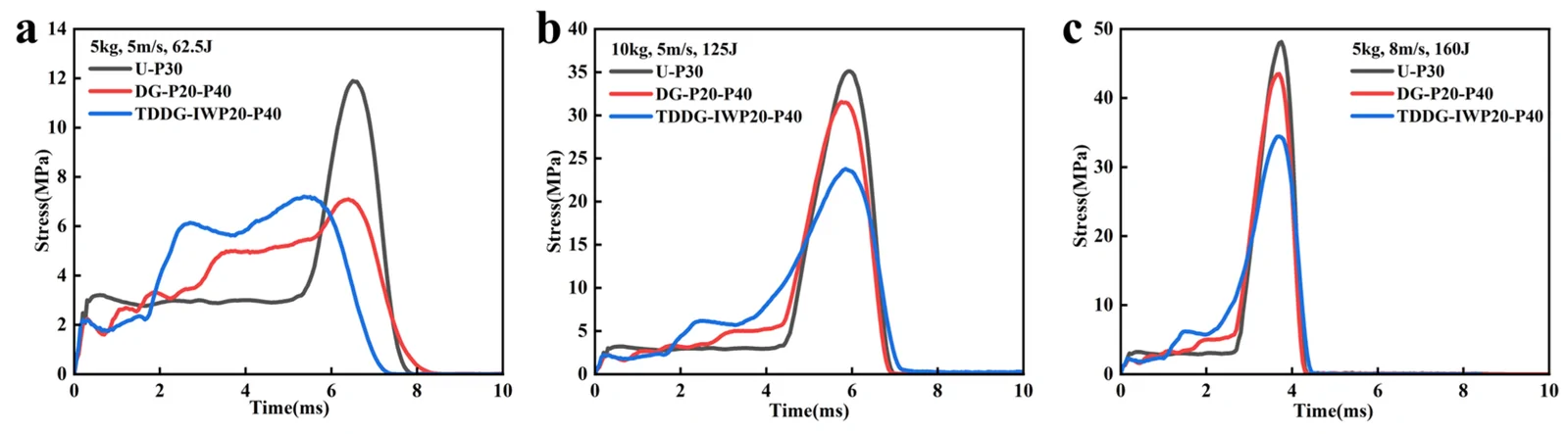
Nominal impact stress–time histories of U-P30, DG-P20-P40, and TDDG-IWP20-P40 under three impact conditions: (**a**) 5 kg and 5 m/s (62.5 J); (**b**) 10 kg and 5 m/s (125 J); and (**c**) 5 kg and 8 m/s (160 J).

Figure 13 further compares the nominal impact stress–time histories under the three impact conditions. At 62.5 J, the peak impact stresses of U-P30, DG-P20-P40, and TDDG-IWP20-P40 were 11.89, 7.09, and 7.19 MPa, respectively. Relative to U-P30, DG-P20-P40 and TDDG-IWP20-P40 reduced the peak stress by 40.4% and 39.5%, respectively. The peak stress of TDDG-IWP20-P40 was only 1.4% higher than that of DG-P20-P40, indicating that the two graded designs provided essentially comparable load mitigation under the lower-energy condition. At 125 J, TDDG-IWP20-P40 exhibited a clearer advantage, reducing the peak stress by 32.2% relative to U-P30 and by 24.6% relative to DG-P20-P40. Under the 160 J condition, the peak stresses were 48.14, 43.50, and 34.41 MPa, respectively, and TDDG-IWP20-P40 achieved reductions of 28.5% and 20.9% relative to U-P30 and DG-P20-P40. Overall, TDDG-IWP20-P40 consistently reduced the peak impact stress relative to the uniform U-P30 structure across all three investigated conditions. Its response was comparable to that of DG-P20-P40 at 62.5 J, while the additional load-mitigation benefit of coupled topology–density grading became clearly evident at 125 and 160 J. These results demonstrate that the peak-stress mitigation of TDDG-IWP20-P40 is not specific to the reference 125 J condition, although the magnitude of its advantage depends on the impact condition.

It should be emphasized that these impact results are comparative numerical predictions under the adopted rate-independent, elastic-perfectly plastic PLA model rather than experimentally validated dynamic performance. Their quantitative magnitudes may differ in physical impact tests because strain-rate sensitivity, damage/failure, and printing-induced anisotropy were not explicitly represented.

### 4.4. Deformation Evolution and Load-Mitigation Mechanisms

**Figure 14:**
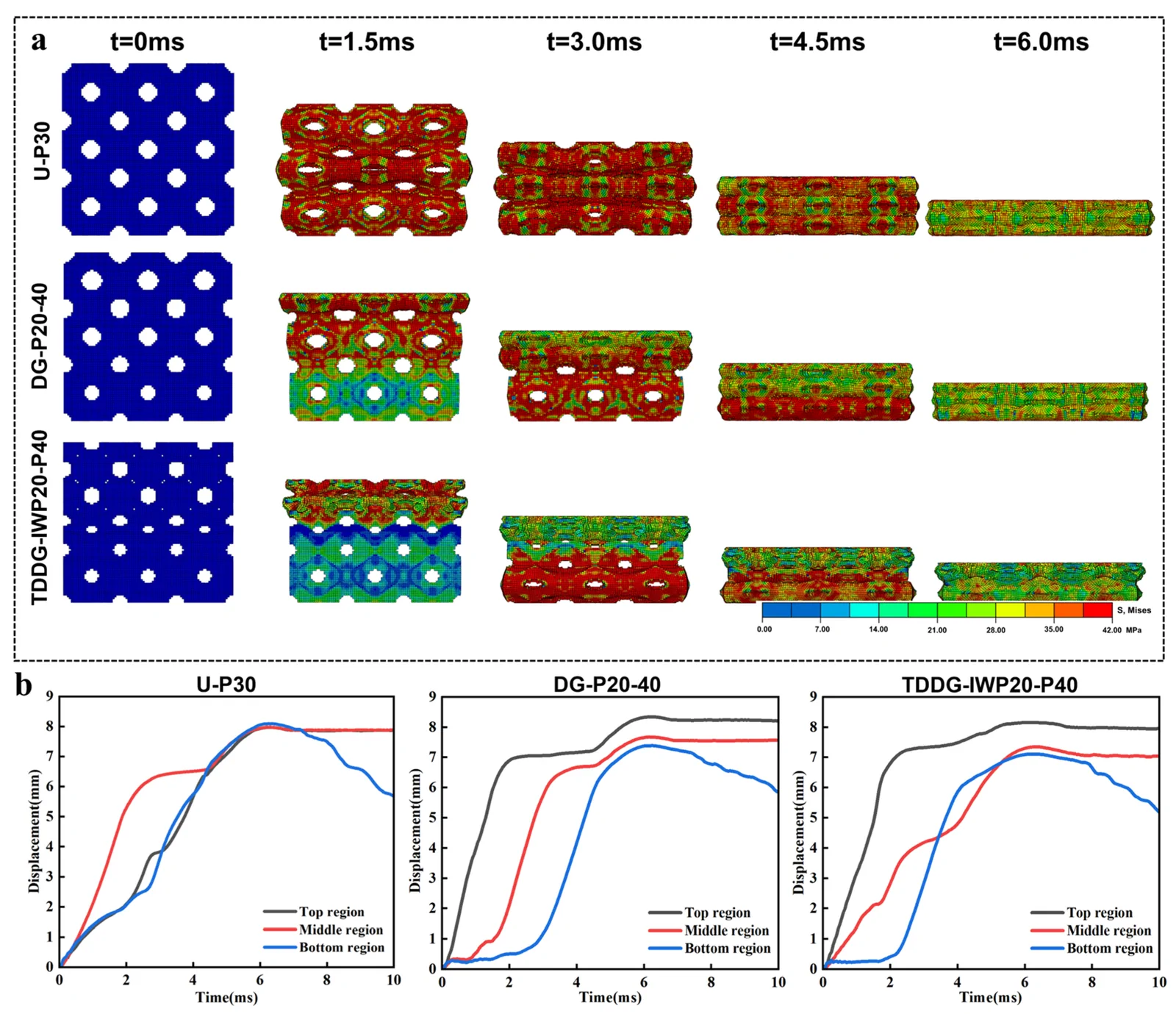
Deformation evolution and quantitative crushing progression of the three TPMS energy absorbers under impact loading: (**a**) deformation modes and von Mises stress distributions at selected impact times; (**b**) compression-displacement histories of the top, middle, and bottom regions of U-P30, DG-P20-P40, and TDDG-IWP20-P40.

The differences in the impact responses presented in Section 4.3 can be explained by the deformation sequences shown in Figure 14. Although the three structures absorbed similar amounts of impact energy, their deformation localization and progression toward densification differed significantly, leading to distinct peak-stress responses.

For U-P30, the uniform topology and relative density resulted in a nearly uniform distribution of collapse resistance. Thus, multiple regions entered the advanced crushing and densification stages within a relatively short interval. The rapid exhaustion of the available void space caused a sharp increase in structural resistance, producing the highest peak impact stress of 35.105 MPa.

For DG-P20-P40, the increasing relative density promoted sequential deformation from the low-density impact-facing region toward the high-density support-facing region. The low-density region deformed first, while the denser region was engaged progressively as compression proceeded. This delayed the onset of global densification and distributed energy absorption over a longer period, reducing the peak impact stress to 31.544 MPa. However, because the P topology was retained throughout the structure, the improvement was governed primarily by density grading.

TDDG-IWP20-P40 exhibited a more effective staged crushing response through the coordinated variation in topology and relative density. The low-density IWP region initiated deformation at a relatively low loading level and dissipated energy during the early stage of impact. The sigmoid transition region then enabled a gradual transfer of load toward the denser P region, avoiding an abrupt change in collapse resistance. During the later stage, the P40 region provided increasing support while retaining sufficient deformation capacity. This sequential engagement reduced the simultaneous densification of different regions and allowed more kinetic energy to be dissipated before final compaction.

As a result, TDDG-IWP20-P40 redistributed energy absorption over a longer deformation interval and reduced the kinetic energy remaining at the final densification stage. This mechanism produced a smoother impactor deceleration, extended the impact duration to 7.60 ms, and reduced the peak stress to 23.786 MPa—32.2% lower than that of U-P30 and 24.6% lower than that of DG-P20-P40. Therefore, the primary advantage of the TDDG design lies in regulating the spatial and temporal sequence of crushing rather than increasing the total amount of absorbed energy.

To further quantify the crushing progression along the specimen height, each absorber was divided into three equal regions along the impact direction: the top impact-facing region, the middle region, and the bottom support-facing region. The compression displacement of each region was extracted during the impact process and compared in Figure 14. For U-P30, the three regions deformed within a relatively overlapping time interval, indicating a more global crushing mode. In contrast, DG-P20-P40 and TDDG-IWP20-P40 exhibited clearer staged crushing behavior. In TDDG-IWP20-P40, the low-density IWP-dominated top region deformed first, while the middle transition region and the bottom P-dominated support region remained less deformed during the early stage and were engaged progressively as compression proceeded. This sequential engagement reduced the initial structural resistance and avoided the simultaneous densification of multiple regions, thereby suppressing the rapid rise in impact stress while maintaining comparable energy absorption.

### 4.5. Aerospace-Oriented Conceptual Buffer Design and Study Limitations

**Figure 15:**
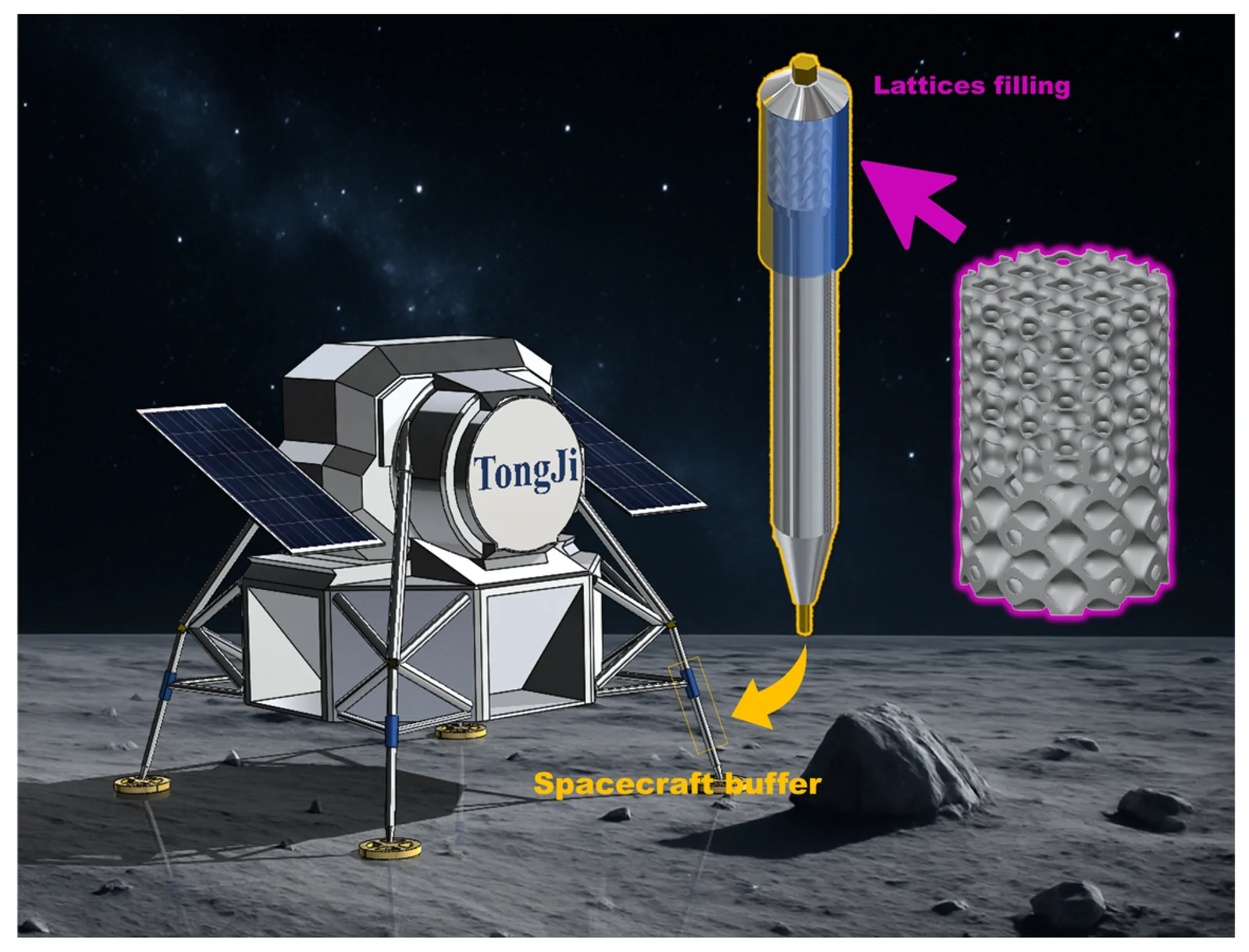
Conceptual cylindrical buffer core incorporating the TDDG-IWP20-P40 architecture for potential spacecraft landing applications.

Based on the load-mitigation mechanism identified in Section 4.3 and Section 4.4, the TDDG-IWP20-P40 architecture was extended to a conceptual cylindrical buffer core for spacecraft landing applications, as illustrated in Figure 15. This design demonstrates the geometric transfer of the proposed architecture from a cubic specimen to a more application-oriented component. The cylindrical core retains the same impact-to-support arrangement and the coordinated gradients in topology and relative density.

The impact-facing region consists of low-density IWP-dominated cells to initiate deformation at a relatively low loading level. The architecture then transitions smoothly through the sigmoid interpolation region toward the high-density P-dominated support-facing region, which provides increasing resistance during the later stage of compression. This arrangement is intended to promote progressive crushing, delay global densification, and reduce the peak load transmitted to the protected structure.

The conceptual cylindrical design demonstrates the geometric adaptability of the implicit TPMS modeling framework. Its dimensions, density range, transition position, gradient sharpness, and topology distribution could be tailored to different impact-energy, allowable-load, and installation-space requirements. Although the cylindrical component may exhibit some quantitative differences from the cubic specimens because of changes in external geometry and boundary conditions, the underlying topology–density grading strategy and progressive-crushing mechanism remain applicable.

The present study has several limitations. The constituent topologies were selected based on quasi-static compression tests, while the dynamic responses were evaluated numerically under three selected combinations of impact mass and velocity and a single axial loading direction. No experimental impact validation was performed. In addition, the adopted rate-independent, elastic-perfectly plastic PLA model does not explicitly account for strain-rate sensitivity, hardening, damage/failure, or printing-induced anisotropy. Consequently, the absolute values of the predicted peak stress and SEA may differ from those obtained in physical impact tests, and the reported impact results should be interpreted as comparative numerical predictions rather than fully validated dynamic performance. Manufacturing defects, geometric size effects, repeated impacts, oblique loading, and environmental conditions were also not comprehensively considered.

Accordingly, Figure 15 should be regarded as an application-oriented conceptual demonstration rather than a validated spacecraft landing component. Future work will focus on impact testing of the TDDG specimens and corresponding reference structures, broader impact-condition and gradient-parameter studies, and component- and system-level simulations under representative landing conditions.

## 5. Conclusions

This study proposed a voxel-based topology–density dual-gradient (TDDG) TPMS absorber for impact-load mitigation. An integrated framework combining implicit TPMS modeling, STL-to-INP voxelization, quasi-static constituent screening, and explicit impact simulation was established. The main conclusions are as follows:

The voxel-based C3D8R model achieved mechanical predictions comparable to those of the C3D4 tetrahedral model while reducing the wall-clock time from 4222 to 497 s. This confirms the suitability of voxelization for the efficient finite element analysis of geometrically complex TPMS structures.

Quasi-static screening revealed distinct density-dependent advantages among the investigated topologies. IWP20 provided a low initial peak stress, a large densification strain, and favorable SEA, whereas P40 maintained a longer effective compression stroke and achieved the highest SEA among the nine uniform configurations. Their complementary characteristics supported the selection of IWP20 and P40 as the impact-facing and support-facing constituents, respectively.

Under the adopted PLA material assumptions, explicit simulations at an impact energy of 125 J showed that TDDG-IWP20-P40 reduced the peak impact stress by 32.2% relative to U-P30 and by 24.6% relative to DG-P20-P40, while maintaining a comparable SEA. The improvement was attributed to sequential crushing from the low-density IWP region to the high-density P region, which redistributed energy dissipation over a longer interval and suppressed the stress rise associated with final densification. Additional simulations at 62.5 and 160 J confirmed that the peak-stress mitigation relative to U-P30 persisted across the investigated impact conditions, while the additional advantage over DG-P20-P40 became more pronounced with increasing impact severity.

Overall, the proposed TDDG strategy provides an effective approach for regulating impact loads without sacrificing energy-absorption capacity. The conceptual cylindrical buffer demonstrates its potential for aerospace applications, although further impact experiments and component-level validation are required before practical implementation.

## Figures and Tables

**Table 1:** Topology–density screening matrix for the quasi-static compression study.

Topology	Target Relative Density	Model Designation
P	20%, 30%, 40%	P20, P30, P40
G	20%, 30%, 40%	G20, G30, G40
IWP	20%, 30%, 40%	IWP20, IWP30, IWP40

**Table 2:** Voxelization accuracy and computational cost of a single P20 unit cell.

Resolution	15	20	30	40
Voxelized Model	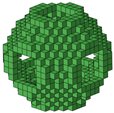	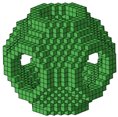	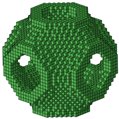	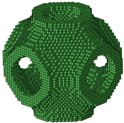
Element number	648	1552	5340	12,724
Voxel size (mm)	0.66 mm	0.50 mm	0.33 mm	0.25 mm
Volume (mm^3^)	192.00	194.00	197.78	198.81
Relative density	19.200%	19.400%	19.778%	19.881%
Density error	4.000%	3.000%	1.110%	0.595%
Wall time (s)	222	284	497	873

**Table 3:** Quasi-static performance metrics obtained from tests of the nine uniform TPMS configurations.

Model	Elastic Modulus (MPa)	Initial Peak Stress (MPa)	Densification Strain (%)	SEA (J/g)	CFE (%)
P20	32.344	1.336	51.78	2.395	71.92
P30	76.797	3.151	55.38	4.287	80.24
P40	132.082	5.396	49.16	5.213	86.79
G20	39.001	1.617	44.95	2.975	85.31
G30	76.0323	3.448	42.22	4.091	90.96
G40	128.794	5.413	38.84	4.286	87.76
IWP20	28.424	1.299	54.67	3.079	85.33
IWP30	70.639	3.261	55.04	5.496	81.67
IWP40	137.217	5.581	30.28	3.994	80.53

**Table 4:** Impact performance evaluation metrics of the three TPMS energy absorbers.

Model	Peak Impact Stress (MPa)	Maximum Compressive Strain (%)	SEA (J/g)	Impact Duration (ms)
U-P30	35.105	79.95	12.287	7.05
DG-P20-40	31.544	77.86	12.151	7.25
TDDG-IWP20-P40	23.786	75.02	12.223	7.60

## Data Availability

The raw data supporting the conclusions of this article will be made available by the authors upon request.

## Abbreviations

The following abbreviations are used in this manuscript:

TPMS	Triply periodic minimal surface
P	Schwarz primitive
G	Gyroid
IWP	I-graph and wrapped package
*PCF*	The peak compression force
SEA	The specific energy absorption
*MCF*	The mean crushing force
*CFE*	The crushing force efficiency
U-	uniform
DG-	density-graded
TDDG-	Topology–density dual-gradient

## Appendix A

### Appendix A.1. Additional Voxel-Size Verification

To further verify the applicability of the selected 0.33 mm voxel size to TPMS geometries other than P20, additional convergence comparisons were performed for the IWP20 and TDDG-IWP20-P40 structures using voxel sizes of 0.33 and 0.25 mm.

IWP20 was selected as a conservative uniform-topology case because it has the smallest characteristic wall thickness among the TPMS structures considered in this study. As shown in Figure A1a, the quasi-static force–displacement responses obtained using the two voxel sizes are in close agreement in terms of initial stiffness, peak compression force, and overall crushing behavior.

The voxel-size sensitivity of TDDG-IWP20-P40 was further evaluated under the 125 J impact condition. Figure A1b compares the nominal stress–strain responses obtained using voxel sizes of 0.33 and 0.25 mm. The two resolutions produce comparable peak impact stresses and overall response curves. The corresponding quantitative results are summarized in Table A1. The relative difference was calculated using the 0.25 mm model as the reference:

$$\mathrm{Relative}\;\mathrm{difference}=\left|X_{0.33}-X_{0.25}\right|/X_{0.25}\times 100\%$$

**Table A1:** Quantitative comparison of the IWP20 and TDDG-IWP20-P40 responses obtained using voxel sizes of 0.33 and 0.25 mm.

Structure	Response Metric	0.33 mm	0.25 mm	Relative Difference
IWP20	Peak force (N)	207.88	213.32	2.55%
	SEA (J/g)	4.81	4.40	9.32%
TDDG-IWP20-P40	Peak impact stress (MPa)	23.786	23.566	0.93%
	SEA (J/g)	12.22	12.29	0.57%

These results demonstrate that further refinement from 0.33 to 0.25 mm produces only minor changes in the global quasi-static and impact responses. Therefore, the selected 0.33 mm voxel size provides an appropriate balance between numerical accuracy and computational efficiency for the IWP20 and TDDG-IWP20-P40 structures.

### Appendix A.2. Numerical Energy Balance of the Explicit Impact Simulation

The numerical energy balance of the TDDG-IWP20-P40 model under the 125 J impact condition was examined to evaluate the stability of the Abaqus/Explicit simulation involving C3D8R elements, severe deformation, self-contact, and densification. The monitored whole-model energy components included kinetic energy (ALLKE), internal energy (ALLIE), total energy (ETOTAL), artificial strain energy (ALLAE), and frictional dissipation energy (ALLFD).

As shown in Figure A2, the initial kinetic energy was approximately 125 J, consistent with the prescribed impactor mass of 10 kg and initial velocity of 5 m/s. During crushing, the decrease in kinetic energy was accompanied by a progressive increase in internal energy. ETOTAL remained nearly constant throughout the simulation, with a maximum deviation of 1.75% from its initial value, indicating stable overall energy conservation.

ALLAE increased gradually during crushing and reached approximately 15.72 J at the end of the simulation, corresponding to approximately 11% of ALLIE. This contribution was associated mainly with the numerical stabilization of the reduced-integration C3D8R elements during severe deformation, self-contact, and final-stage densification. Although this contribution was not negligible, it remained substantially lower than ALLIE and did not dominate the global energy response. ALLFD also remained lower than ALLIE throughout the impact process. Together with the voxel-size convergence results presented in Appendix A.1, these energy histories support the numerical stability of the global impact-response predictions.
